# A global multicohort study to map subcortical brain development and cognition in infancy and early childhood

**DOI:** 10.1038/s41593-023-01501-6

**Published:** 2023-11-23

**Authors:** Ann M. Alex, Fernando Aguate, Kelly Botteron, Claudia Buss, Yap-Seng Chong, Stephen R. Dager, Kirsten A. Donald, Sonja Entringer, Damien A. Fair, Marielle V. Fortier, Nadine Gaab, John H. Gilmore, Jessica B. Girault, Alice M. Graham, Nynke A. Groenewold, Heather Hazlett, Weili Lin, Michael J. Meaney, Joseph Piven, Anqi Qiu, Jerod M. Rasmussen, Annerine Roos, Robert T. Schultz, Michael A. Skeide, Dan J. Stein, Martin Styner, Paul M. Thompson, Ted K. Turesky, Pathik D. Wadhwa, Heather J. Zar, Lilla Zöllei, Gustavo de los Campos, Rebecca C. Knickmeyer, the ENIGMA ORIGINs group

**Affiliations:** 1 Institute for Quantitative Health Sciences and Engineering, Michigan State University, East Lansing, MI USA; 2 Departments of Epidemiology & Biostatistics, Michigan State University, East Lansing, MI USA; 3 Mallinickrodt Institute of Radiology, Washington University School of Medicine, St. Louis, MO USA; 4 Department of Medical Psychology, Charité–Universitätsmedizin Berlin, corporate member of Freie Universität Berlin and Humboldt-Universität zu Berlin, Berlin, Germany; 5 Department of Pediatrics, University of California Irvine, Irvine, CA USA; 6 Development, Health and Disease Research Program, University of California Irvine, Irvine, CA USA; 7 Department of Obstetrics and Gynaecology, Yong Loo Lin School of Medicine, National University of Singapore, Singapore, Singapore; 8 Singapore Institute for Clinical Sciences, Agency for Science, Technology and Research, Singapore, Singapore; 9 Department of Radiology, University of Washington Medical Center, Seattle, WA USA; 10 Division of Developmental Paediatrics, Department of Paediatrics and Child Health, Red Cross War Memorial Children’s Hospital, University of Cape Town, Cape Town, South Africa; 11 Neuroscience Institute, University of Cape Town, Cape Town, South Africa; 12 Masonic Institute for the Developing Brain, University of Minnesota Medical School, Minneapolis, MN USA; 13 Department of Diagnostic & Interventional Imaging, KK Women’s and Children’s Hospital, Singapore, Singapore; 14 Harvard Graduate School of Education, Harvard University, Cambridge, MA USA; 15 Department of Psychiatry, University of North Carolina at Chapel Hill, Chapel Hill, NC USA; 16 Carolina Institute for Developmental Disabilities, University of North Carolina at Chapel Hill, Carboro, NC USA; 17 Department of Psychiatry, Oregon Health & Science University, Portland, OR USA; 18 South African Medical Research Council (SA-MRC) Unit on Child & Adolescent Health, University of Cape Town, Cape Town, South Africa; 19 Department of Psychiatry, University of Cape Town, Cape Town, South Africa; 20 Department of Paediatrics and Child Health, University of Cape Town, Faculty of Health Sciences, Cape Town, South Africa; 21 Department of Radiology, University of North Carolina at Chapel Hill, Chapel Hill, NC USA; 22 Department of Biomedical Engineering, National University of Singapore, Singapore, Singapore; 23 NUS (Suzhou) Research Institute, National University of Singapore, Suzhou, China; 24 The N.1 Institute for Health, National University of Singapore, Singapore, Singapore; 25 Institute of Data Science, National University of Singapore, Singapore, Singapore; 26 Department of Biomedical Engineering, Johns Hopkins University, Baltimore, MD USA; 27 Department of Health Technology and Informatics, Hong Kong Polytechnic University, Hung Hom, China; 28 SAMRC Unit on Risk and Resilience in Mental Disorders, Department of Psychiatry, University of Cape Town, Cape Town, South Africa; 29 Center for Autism Research, Children’s Hospital of Philadelphia and the University of Pennsylvania, Philadelphia, PA USA; 30 Research Group Learning in Early Childhood, Max Planck Institute for Human Cognitive and Brain Sciences, Leipzig, Germany; 31 Department of Computer Science, University of North Carolina at Chapel Hill, Chapel Hill, NC USA; 32 Imaging Genetics Center, Stevens Neuroimaging & Informatics Institute, Keck School of Medicine of University of Southern California, Marina del Rey, CA USA; 33 Departments of Psychiatry and Human Behavior, Obstetrics & Gynecology, Epidemiology, University of California, Irvine, Irvine, CA USA; 34 A.A. Martinos Center for Biomedical Imaging, Massachusetts General Hospital, Harvard Medical School, Charlestown, MA USA; 35 Department of Statistics & Probability, Michigan State University, East Lansing, MI USA; 36 Department of Pediatrics and Human Development, Michigan State University, East Lansing, MI USA

**Keywords:** Development of the nervous system, Psychology, Magnetic resonance imaging, Behavioural methods

## Abstract

The human brain grows quickly during infancy and early childhood, but factors influencing brain maturation in this period remain poorly understood. To address this gap, we harmonized data from eight diverse cohorts, creating one of the largest pediatric neuroimaging datasets to date focused on birth to 6 years of age. We mapped the developmental trajectory of intracranial and subcortical volumes in ∼2,000 children and studied how sociodemographic factors and adverse birth outcomes influence brain structure and cognition. The amygdala was the first subcortical volume to mature, whereas the thalamus exhibited protracted development. Males had larger brain volumes than females, and children born preterm or with low birthweight showed catch-up growth with age. Socioeconomic factors exerted region- and time-specific effects. Regarding cognition, males scored lower than females; preterm birth affected all developmental areas tested, and socioeconomic factors affected visual reception and receptive language. Brain–cognition correlations revealed region-specific associations.

## Abstract (web summary)

The ENIGMA-ORIGINs group presents a large and globally diverse pediatric neuroimaging dataset from birth to age 6. They use this resource to study the effects of sociodemographics and adverse birth outcomes on trajectories of brain volumes and cognition.

## Main

Early childhood (birth to 6 years) represents a dynamic and critical period in human brain development. At a cellular level, this period is marked by glial proliferation and migration, dendritic arborization, synaptogenesis, myelination, programmed cell death and synaptic and axonal elimination^1^. At the cognitive and behavioral levels, several abilities, including language, memory, social cognition, emotional regulation and executive function, emerge and elaborate. Sensory and motor skills also develop rapidly^2^. Between the cellular and cognitive/behavioral levels lie macroscale brain properties that are best characterized by imaging-based phenotypes (for example, global, regional and subcortical volumes, cortical thickness, white matter diffusivity, functional connectivity and so on). Our understanding of early development of these brain-related phenotypes has increased substantially in the past 20 years, driven by an expanding number of cross-sectional and longitudinal neuroimaging studies, particularly magnetic resonance imaging (MRI) studies^3,4^. However, many knowledge gaps remain, including (1) very few large-scale studies have mapped the development of brain-related phenotypes from birth to 6 years of age in diverse global populations with dense data, (2) there is limited information on how sociodemographic factors and adverse birth outcomes shape neurodevelopmental trajectories, and (3) the neural correlates of variations in cognitive development are not fully understood. The present study seeks to address these gaps with a particular focus on the development of intracranial volume (ICV) and subcortical structures, including the thalamus, hippocampus, amygdala, caudate, putamen and globus pallidus.

Regarding the first knowledge gap, most studies on ICV and subcortical volume development focus on later childhood, adolescence and adulthood, with limited data below 6 years of age. Studies focusing specifically on early life generally have narrow time frames, particularly birth to age 2 (ref. ^4^). Furthermore, published research on subcortical volume trajectories in early childhood is often limited by cross-sectional study design, low sample size, discontinuous age ranges and/or a lack of diversity in participants^5^. Longitudinal studies are necessary to model individual developmental trajectories and to elucidate how parameters of such trajectories are influenced by birth outcomes and sociodemographic factors^6^. Also, small sample size (leading to underpowered studies) and limited racial and socioeconomic diversity reduce reproducibility and replicability of published results. The study by Marek et al. points out that brain-wide association studies require thousands of participants to accurately identify brain–phenotype associations^7^. Collaborative research overcomes these challenges by offering adequately powered study designs and recognizing differences across cultures and measurement methods. Addressing both inter- and intraindividual variability can improve the accuracy of growth curves and increase the generalizability of results^8,9^. The recent work on brain charts was one of the largest studies to map normative brain growth across the lifespan^10^ and a step in the direction of inclusion of diverse global populations; the current study differs by focusing on specific subcortical structures, examining the influence of socioeconomic factors (SES) and adverse birth outcomes and examining brain–cognition associations.

Regarding the second knowledge gap, as with studies of age-related change in ICV and subcortical volumes, studies associating adverse birth outcome and sociodemographic factors with brain structure mainly focused on brain outcomes characterized in late childhood, adolescence and adulthood. Early childhood, the period when the brain is most malleable to environmental effects^11^, has not been studied as thoroughly due to practical and technical challenges of conducting imaging studies in this age range. Ample studies show that sex^12,13^, adverse birth outcomes (preterm birth and low birthweight^14^) and SES (family income and maternal education^15^) contribute to variation in structural brain development and cognition in late childhood and adolescence. Understanding the effects of these factors on neuroanatomical development in early childhood may reveal the earliest deviations from typical trajectories and provide guidance for interventions to prevent or reverse adverse neurodevelopment in children at risk^16^.

Regarding the third knowledge gap, given the dynamic nature of brain development in early childhood and concurrent development of cognitive and behavioral characteristics, it is important to understand the neural basis of cognitive abilities across this age range. ICV and subcortical structures play important roles in cognition^17,18^, but our understanding of the neuroanatomical correlates of early cognitive development is limited. Cognitive functioning in childhood predicts later cognitive competence^19^, and a better understanding of its neuroanatomical correlates could help inform interventions to support early cognitive development.

The Organization for Imaging Genomics in Infancy (ORIGINs) was established to address the gaps discussed above and to facilitate large-scale genetics studies of brain structure and function during infancy and early childhood. ORIGINs is a working group within the Enhancing Neuroimaging Genetics through Meta‐Analysis (ENIGMA) Consortium, a global network of greater than 2,025 scientists studying the human brain in health and disease^20^. Here, we introduce one of the largest pediatric neuroimaging datasets spanning birth through age 6 and use it to map the trajectory of ICV, subcortical structures (thalamus, hippocampus, amygdala, caudate, putamen and pallidum) and cognitive development as indexed by the Mullen Scales of Early Learning (MSEL). We analyzed this unique dataset to investigate the effects of sex, gestational age, birthweight, maternal education and family income on trajectories of ICV and subcortical volumes and on cognitive development. Finally, we examined brain–cognition correlations in this age range. This dataset includes over 2,000 children across eight contributing sites in four countries (Germany, Singapore, South Africa and the United States). This study lays a strong foundation for understanding ICV and subcortical brain development and how it relates to early cognitive development encompassing children from diverse ethnic and socioeconomic backgrounds. This information is important as brain development in these early years establishes the path for lifelong cognitive development and psychiatric risk^4^.

## Results

### Developmental trajectories of ICV and subcortical structures

Demographic details of the whole sample and information regarding sample distributions for each cohort are given in Table 1.

**Table 1:** Demographic distribution of participants

	Sample size	Sex	Gestational age	Birthweight	Maternal education	Family income
	Individuals/observations	Male/female	Preterm/full term	Low/normal	Primary–secondary/tertiary	Low/medium-high/missing
All	2,108/3,607	1,102/1,006	436/1,672	365/1,743	651/1,457	606/1,261/241
%		52.3/47.7	20.7/79.3	17.3/82.7	30.9/69.1	28.8/59.8/11.4
BCP	172/317	80/92	2/170	1/171	5/167	14/154/4
%	8.2/8.8	46.5/53.5	1.2/98.8	1/99	2.9/97.1	8.2/89.5/2.3
Boston (BCH)	130/181	66/64	3/127	6/124	3/127	9/108/13
%	6.2/5	50.8/49.2	2.3/97.7	4.6/95.4	2.3/97.7	6.9/83.1/10
Cape Town (DCHS)	135/135	70/65	9/126	5/130	128/7	45/90
%	6.4/3.7	51.9/48.1	6.7/93.3	3.7/96.3	94.8/5.2	33.3/66.7
EBDS	1,013/2,152	538/475	384/629	319/694	350/663	410/537/66
%	48/59.7	53.1/46.9	37.9/62.1	31.5/68.5	34.6/65.4	40.5/53/6.5
GUSTO	357/421	176/181	21/336	25/332	131/226	68/264/25
%	16.9/11.7	49.3/50.7	5.9/94.1	7/93	36.7/63.3	19/74/7
IBIS	86/186	47/39	1/85	1/85	0/86	12/72/2
%	4.1/5.2	54.7/45.3	1.2/98.8	1.2/98.8	0/100	14/83.7/2.3
Max Planck	127/127	73/54	10/117	4/123	5/122	0/0/127
%	6/3.5	57.5/42.5	7.9/92.1	3.1/96.9	3.9/96.1	0/0/100
UCI	88/88	52/36	6/82	4/84	29/59	48/36/4
%	4.2/2.4	59/41	6.8/93.2	4.5/95.5	33/67	54.6/40.9/4.5

BCP, Baby Connectome Project; Boston (BCH), Boston Children’s Hospital; Cape Town (DCHS), Drakenstein Child Health Study; GUSTO, Growing Up in Singapore Towards Healthy Outcomes, Singapore; IBIS, Infant Brain Imaging Study Network; UCI, University of California, Irvine.

**Fig. 1:**
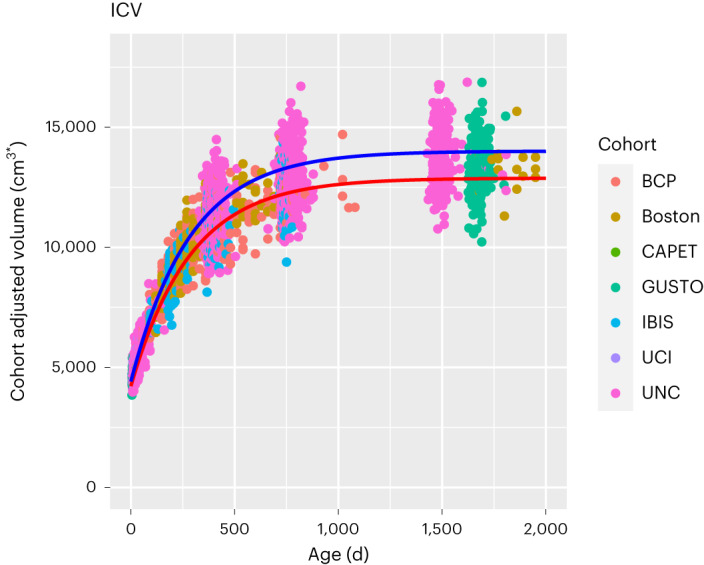
The effect of sex on the developmental trajectory of ICV. The lines represent the fitted growth curve for the male and female sex from the nonlinear mixed model regression (number of individuals = 1,835, number of observations = 3,168). Males (blue line) have significantly larger ICV than females (red line) throughout the age range studied (the *P* values derived from two-sided *t*-tests are 1.68 × 10^–12^ at intercept and 1.88 × 10^–60^ at asymptote; the Bonferroni-corrected *P* value threshold for significance was <0.0001). Circles represent individual data points, and colors represent the cohorts; BCP, Baby Connectome Project; Boston, Boston Children’s Hospital/Harvard Medical School; CAPET, Drakenstein Child Health Study, Cape Town; GUSTO, Growing Up in Singapore Towards Healthy Outcomes, Singapore; IBIS, Infant Brain Imaging Study Network; UCI, University of California, Irvine; UNC, University of North Carolina Early Brain Development Study.

To map longitudinal brain development, we fitted (mixed-effects) subject-specific nonlinear longitudinal growth curves to ICV and subcortical structures (thalamus, amygdala, hippocampus, caudate, putamen and pallidum). Our growth curve models have subject-specific intercepts (that is, volume at birth), asymptote and growth rate parameters. In our hierarchical model, we included effects of birth outcomes and sociodemographic factors on intercepts and asymptotes and random effects of cohort and subject. The fitted growth curves for male and female ICV are displayed in Fig. 1. Growth curves for ICV and subcortical structures as they relate to each predictor variable are found in Supplementary Figs. 2–6.

Our results show that ICV and subcortical structures follow nonlinear growth patterns from birth to age 6, with maturation ages differing across regions. We define maturation age as the age at which regional volume reaches 99% of its asymptotic value. We find that ICV matures by age 3.3–3.5 years. Among subcortical structures, the amygdala matures earliest (3.3 years), followed by the hippocampus (4.6 years), putamen (4.6 years), globus pallidus (5 years) and caudate (5.5 years). The thalamus has the most protracted trajectory, reaching maturation by 7.3–7.5 years.

Sex, adverse birth outcomes and SES influence global and regional developmental trajectories (Table 2). Males show significantly larger volume throughout the age range for all structures. Children born preterm had significantly lower ICV and amygdala, hippocampus and thalamus volumes at birth. However, the effect was no longer significant at asymptote, suggesting that volumes catch up as the child matures. Similarly, for children with low birthweight, all volumes were significantly lower at birth. This effect was also non-significant by asymptotic age.

**Table 2:** Birth outcomes and sociodemographic factors have significant effects on structural brain development^a^

			ICV	Thalamus	Hippocampus	Amygdala	Caudate	Putamen	Globus pallidus
**Reference curve**									
Reference^b^	Intercept	Effect	402,952.30	8,924.17	2,190.29	398.78	3,711.22	3,345.64	1,141.27
		(s.e.)	(2,676.98)	(42.15)	(13.16)	(5.80)	(22.54)	(51.25)	(16.79)
	Asymptote	Effect	1,285,208.00	13,292.35	5,159.63	2,082.93	6,947.72	8,535.24	2,114.39
		(s.e.)	(6,309.91)	(80.24)	(31.85)	(13.30)	(53.36)	(55.41)	(15.88)
	Growth Rate	Effect	−5.66	−6.64	−6.02	−5.60	−6.26	−6.01	−6.16
		(s.e.)	(0.01)	(0.02)	(0.01)	(0.01)	(0.02)	(0.01)	(0.03)
**Main effects**									
Male sex	Intercept	Effect	19,177.04**	423.13**	80.27**	19.84**	114.07**	226.31**	40.48*
		(s.e.)	(2,690.45)	(42.57)	(13.34)	(5.48)	(21.93)	(50.16)	(13.33)
		%	4.76	4.74	3.66	4.98	3.07	6.76	3.55
	Asymptote	Effect	112,624.10**	990.70**	365.18**	190.05**	325.09**	671.36**	182.94**
		(s.e.)	(6,517.97)	(74.91)	(32.32)	(13.37)	(53.41)	(59.57)	(16.34)
		%	8.76	7.35	7.07	9.12	4.67	7.86	8.63
Preterm birth	Intercept	Effect	−48,142.85**	−282.32**	−179.79**	−109.84**	−82.43*	−218.48*	−71.87*
		(s.e.)	(4,056.34)	(67.89)	(20.14)	(8.86)	(34.63)	(87.78)	(22.75)
		%	−11.95	−3.16	−8.2	−27.54	−2.22	−6.53	−6.3
	Asymptote	Effect	17,063.40	−184.55	−93.51	−41.81*	−143.08	−56.33	−34.43
		(s.e.)	(9,981.36)	(112.09)	(47.82)	(19.84)	(79.84)	(89.71)	(23.57)
		%	1.33	−1.46	−1.83	−2	−2.1	−0.67	−1.65
Low birthweight	Intercept	Effect	−45,116.71**	−295.56**	−176.08**	−74.96**	−185.32**	−385.05**	−96.05**
		(s.e.)	(4,263.44)	(69.66)	(20.97)	(9.16)	(35.98)	(90.67)	(24.03)
		%	−11.2	−3.31	−8.04	−18.8	−4.99	−11.5	−8.42
	Asymptote	Effect	−26,109.07*	−110.19	−87.64	−24.73	−146.68	−165.53	−69.30*
		(s.e.)	(10,355.73)	(115.98)	(49.65)	(20.63)	(82.80)	(93.09)	(24.47)
		%	−2.03	−0.93	−1.71	−1.19	−2.14	−1.96	−3.3
Low maternal education	Intercept	Effect	−16,269.64**	−186.31*	−4.71	7.27	−120.10**	383.86**	205.47**
		(s.e.)	(3,203.33)	(55.66)	(16.63)	(6.52)	(24.50)	(70.81)	(17.43)
		%	−4.04	−2.09	−0.22	1.82	−3.24	11.47	18
	Asymptote	Effect	−45,624.15**	−394.91**	−106.46	−88.94**	−298.09**	−260.66*	−92.64**
		(s.e.)	(8,944.40)	(101.29)	(44.70)	(18.62)	(71.94)	(84.23)	(22.38)
		%	−3.55	−2.94	−2.06	−4.27	−4.28	−3.03	−4.26
Low-income family	Intercept	Effect	4,526.54	−92.39	−58.86**	−13.37*	10.61	−254.62**	−51.61**
		(s.e.)	(3,174.30)	(53.61)	(16.10)	(6.46)	(25.67)	(60.30)	(14.59)
		%	1.12	−1.04	−2.69	−3.35	0.29	−7.61	−4.52
	Asymptote	Effect	−14,994.01	−398.43**	−162.85**	−45.89*	−233.97**	−336.27**	−85.68**
		(s.e.)	(8,452.62)	(95.18)	(41.57)	(17.25)	(68.13)	(77.43)	(20.71)
		%	−1.17	−2.92	−3.16	−2.21	−3.34	−3.95	−4.1
Number of observations		3,168	3,276	3,242	3,257	3,270	2,245	2,254	
Number of individuals		1,835	1,865	1,857	1,861	1,865	1,469	1,469	

*P < 0.05; **P < 0.0007.

^a^Data were analyzed by two-tailed *t*-test with a Bonferroni post hoc test for *P* value significance. Exact P values are provided in Extended Data Table 1.

^b^Reference: female, full term, normal birthweight, tertiary maternal education and medium high-income family.

Low maternal education was associated with lower ICV and caudate volume across the age range and with thalamus and amygdala volume at asymptote. For the putamen and globus pallidus, low maternal education was associated with larger volumes at birth and smaller volumes at asymptote. Children from lower-income families had significantly lower hippocampus, putamen and pallidum volumes throughout the age range and lower thalamus and caudate volumes only at the asymptote.

### Sensitivity analyses

We performed three sensitivity analyses and split-sample replication analyses to assess the robustness of predictive values of birth outcomes and SES on brain volume trajectories. First, we excluded family income from the model because of its high correlation with maternal education. Results obtained from this subset confirm those from the full model (Supplementary Table 1). However, hippocampal volume was significantly associated with maternal education in this model. Second, we controlled for interindividual variation in head size^21^ by dividing regional volumes by ICV. The only significant effect in this model was male–female difference on caudate volume, with males having smaller relative caudate volume than females (Supplementary Table 2). Finally, we excluded one twin/sibling from each pair in the University of North Carolina Early Brain Development Study (EBDS) cohort to check if relatedness between participants affected the model results. Results were like those of the main analysis (Supplementary Table 3).

Split-sample replication analysis revealed that sex and birth outcomes had robust associations with brain volumes, with most folds showing the same direction of effect and meeting the Bonferroni threshold for significance (Supplementary Table 4). Results for SES variables were more variable. Direction of effect was generally similar between folds but often did not reach the criteria for significance in both folds. This likely reflects reduced power when dividing the sample and the smaller effect size for SES–brain associations.

### Cognitive and motor development

Next, we examined patterns of cognitive and motor development in these children by fitting linear mixed-effects models with fixed effects of covariates and of cohort and participants’ random effects. To account for possible heterogeneity in scales, we also fitted cohort-specific error variances. As displayed in Table 3, we observed a linear increase (slope *P* values < 0.01) in cognitive scores by age in days. Gross motor scores were significantly lower in children born preterm. Visual reception scores were significantly impacted by all predictor variables, with lower scores observed in males, children born preterm, children of mothers with lower education and children from lower-income families. Fine motor scores were lower in males and in children born preterm. Children born preterm or from lower-income families had significantly lower receptive language scores. Preterm birth was a significant predictor of lower expressive language scores. Further, we tested for an interaction effect of predictor variables with age on cognitive scores. The interaction between age and preterm birth was significant for visual reception and receptive language scores. For visual reception, children born full term and preterm were similar until around 1.5 years of age; subsequently, there was an increasing gap between the two, with children born full term scoring higher. For receptive language, children born preterm scored lower in early life but overtook children born full term around 3.5 years of age. The interaction of maternal education and family income with age was significant for visual reception scores. In both cases, a widening gap between children from lower-SES families and children from higher SES emerged around 1.5 years of age. For gross motor scores, interaction between family income and age was significant. Children from lower-income families had higher scores in early infancy than children from higher-income families and lower scores in late toddlerhood and early childhood (Supplementary Table 5 and Supplementary Figs. 7–9). To test if family relatedness affected our results, we performed a sensitivity analysis removing a twin/sibling from the data and reanalyzed the data using the main model. The results were like those of the main analysis (Supplementary Table 6). Split-sample replication analysis revealed non-significant associations but with similar direction of effect for all significant associations in the main analysis (Supplementary Table 7).

**Table 3:** Demographics, birth outcomes and SES significantly influence cognitive and motor development^a^

	Gross motor	Visual reception	Fine motor	Receptive language	Expressive language
	Estimate (s.e.)	Estimate (s.e.)	Estimate (s.e.)	Estimate (s.e.)	Estimate (s.e.)
**Reference growth model**					
Intercept	7.65	6.13	7.5	5.76	5.52
	(0.18)	(0.2)	(0.15)	(0.2)	(0.2)
Age	0.03**	0.03**	0.03**	0.03**	0.03**
	(<0.01)	(<0.01)	(<0.01)	(<0.01)	(<0.01)
**Main effects**					
Male sex	−0.19	−0.51**	−0.3**	−0.4*	−0.4*
	(0.12)	(0.13)	(0.1)	(0.15)	(0.15)
Preterm birth	−0.92**	−0.88**	−0.91**	−1.32**	−1.28**
	(0.19)	(0.22)	(0.16)	(0.25)	(0.25)
Low birthweight	−0.26	−0.64*	−0.52*	−0.06	−0.18
	(0.2)	(0.23)	(0.17)	(0.26)	(0.26)
Low maternal education	0.16	−0.74**	−0.34*	−0.46	−0.27
	(0.2)	(0.22)	(0.16)	(0.26)	(0.26)
Low family income	0.01	−0.6**	−0.11	−0.8**	−0.53*
	(0.16)	(0.18)	(0.13)	(0.21)	(0.21)
Number of observations	2,384	2,404	2,410	2,472	2,477
Number of individuals	1,209	1,203	1,205	1,222	1,223
Interindividual variation	21.5%	10.37%	6.07%	13.12%	19.05%

*P < 0.05; **P < 0.002.

^a^Data were analyzed by two-tailed *t*-test with a Bonferroni post hoc test for *P* value significance. Exact *P* values are provided in Extended Data Table 2.

### Correlations between brain volumes and cognitive scores

**Fig. 2:**
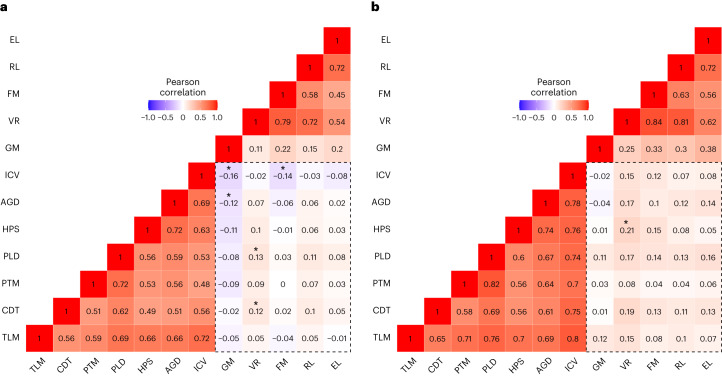
Heat map of the correlation between brain volumes and cognitive scores. **a**,**b**, Correlation assessed between the predicted values for brain volumes and cognitive and motor scores at age 2 using Pearson’s correlations in children born full term (**a**) and preterm (**b**). In full-term children, the significant correlations are ICV–gross motor score (*P* = 0.0000), amygdala–gross motor score (*P* = 0.001), ICV–fine motor score (*P* = 0.0002), caudate–visual reception score (*P* = 0.001) and globus pallidus–visual reception score (*P* = 0.0005). In preterm children, the significant correlation is hippocampus–visual reception score (*P* = 0.0007). Brain volume–cognitive score correlations are highlighted in the dashed rectangle. The squares marked with an asterisk (*) represent significant correlations (*P* < 0.0015, which is the threshold for significance after Bonferroni correction for multiple comparisons). Data were analyzed by two-tailed *t*-tests; TLM, thalamus; CDT, caudate; PTM, putamen; PLD, globus pallidus; HPS, hippocampus; AGD, amygdala; GM, gross motor score; VR, visual reception score; FM, fine motor score; RL, receptive language score; EL, expressive language score.

To assess brain–cognition correlations, we used Pearson’s correlations between predicted brain volumes (ICV and subcortical structures) and cognitive scores at 2 years of age. In the full sample, volumes and cognitive scores showed a slight overall positive correlation. But the direction of effects varied across individual cohorts. Individual cohorts showing positive correlations had a greater representation of children born preterm, whereas those showing negative correlations included few or no children born preterm (Supplementary Table 8). Correlations were then analyzed separately for two subgroups, full-term children and preterm children. For full-term children (Fig. 2a), several brain volumes were significantly correlated with cognitive scores (Supplementary Table 9). ICV was negatively correlated with gross motor and fine motor scores. Amygdala volume was negatively correlated with gross motor scores. Globus pallidus and caudate volumes were positively correlated with visual reception scores. Results were similar when excluding one twin/sibling from each pair (Supplementary Table 10). Split-sample replication analyses revealed that the direction of effect was highly consistent for these associations but seldom met the criteria for significance in both folds (Supplementary Table 11).

For children born preterm (Fig. 2b), most brain volumes were correlated with cognitive scores, with larger volumes associated with higher scores (Supplementary Table 12). However, the only significant relationship was between hippocampal volume and visual reception. This relationship did not meet the significance threshold when excluding one twin/sibling from each pair (Supplementary Table 13). Direction of effect was highly similar across folds in the split-sample analysis but was never significant in both folds (Supplementary Table 14).

To test whether correlations differed between children born full term and those born preterm more than would be expected from sample variability, we computed confidence intervals for the difference in correlation coefficients using a bootstrap method (Supplementary Table 15). Significant differences in correlation coefficients between full-term and preterm children were observed for gross motor scores with thalamus and pallidum volume, fine motor scores with thalamus, caudate, hippocampus, amygdala and ICV and visual reception and expressive language scores with ICV.

**Fig. 3:**
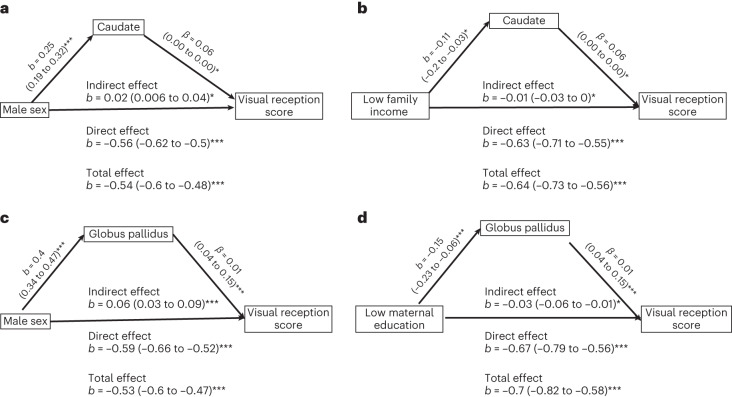
Causal mediation analyses for children born full term. **a**–**d**, Causal mediation analysis reveals partial mediation effects of the caudate and globus pallidus on the association between sociodemographic factors (sex, maternal education and family income) and visual reception scores in children born full term. **a**,**b**, Mediation by the caudate on the effect of sex (**a**; *P* = 0.0052) and low family income (**b**; *P* = 0.015) on visual reception scores (*N* = 760). **c**,**d**, Mediation by the globus pallidus on the effect of sex (**c**; *P* = 0.0004) and low maternal education (**d**; *P* = 0.0012) on visual reception scores (*N* = 659). Bootstrapping was used to estimate *P* values; *P < 0.05; ***P < 0.001. No correction for multiple comparisons was performed.

**Fig. 4:**
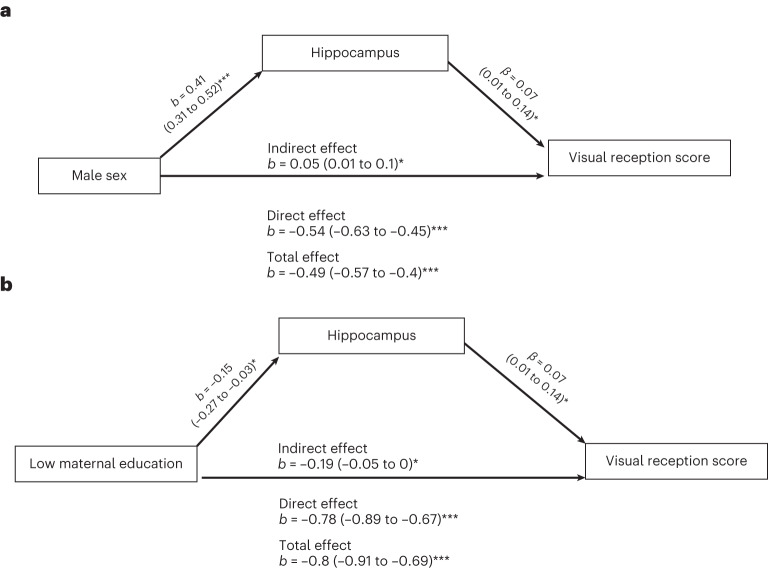
Causal mediation analyses for children born preterm. **a**,**b**, Causal mediation analysis reveals the partial mediation effects of hippocampal volume on the association between sociodemographic factors (sex and maternal education) and visual reception scores in children born preterm. Mediation by the hippocampus on the effect of sex (**a**; *P* = 0.01) and low maternal education (**b**; *P* = 0.041) on visual reception scores (*N* = 269). Bootstrapping was used to estimate *P* values; *P < 0.05; ***P < 0.001. No correction for multiple comparisons was performed.

To explore if the relationships between sociodemographic factors and cognitive and motor development were mediated by brain volumes, we tested the mediation effect on significant brain–cognition correlations using predicted values at age 2 (Supplementary Table 16). In full-term children, the mediation effects by caudate and pallidum volumes on the influence of sex, family income and maternal education on visual reception scores were found to be significant (Fig. 3). In preterm children, the mediation effects of hippocampal volume on the relationship between sociodemographic factors (sex and maternal education) and visual reception scores were found to be significant (Fig. 4). However, the magnitude of mediation effects (indirect effect) was smaller than that of direct effects. Because the effect sizes were so small, we did not conduct split-sample analyses for the mediation models.

## Discussion

Using one of the largest pediatric neuroimaging datasets to date focused on birth to 6 years of age, we addressed three critical questions: (1) What is the trajectory of ICV and subcortical volume development? (2) How do sociodemographic factors and birth outcomes shape neurodevelopmental trajectories? (3) What are the neural correlates of cognitive development? ICV and subcortical structures followed nonlinear growth patterns in early childhood with considerable regional heterogeneity. Developmental trajectories were significantly associated with sex, adverse birth outcomes (preterm birth and low birthweight) and SES (maternal education and family income), and predicted ICV and volumes of the amygdala, hippocampus, caudate and pallidum correlate with different cognitive measures at 2 years of age in children born full term and preterm. Furthermore, associations between cognitive development and sociodemographic factors were partially mediated by subcortical brain volumes with small, but significant, effects.

Regarding the trajectory of ICV and subcortical volumes, we observed a faster growth rate in the first 1,000 postnatal days (∼3 years), followed by a slower growth rate for all volumes. Extremely rapid growth in the first few years of life may make the brains of infants and young children especially vulnerable to environmental insults, such as poverty and preterm birth, and also especially responsive to interventions. As in prior studies of regional brain volumes performed from childhood to adolescence^5^, trajectories of ICV and subcortical structures followed a nonlinear pattern across early childhood, with different structures attaining maturation at different ages. This finding suggests that different regions will have different windows of vulnerability/opportunity.

Among the subcortical structures, maturation age for the amygdala was around 3.3 years. Early structural maturation of the amygdala is consistent with other studies reporting early structural and functional development of the amygdala in primates including humans and suggesting late infancy to childhood as a sensitive period for its development^22^. The amygdala serves important roles in sensing the environment to evaluate potential dangers, process fear and mount an appropriate response^22^. Given the importance of threat stimuli for survival, natural selection may have promoted early development of neural circuits for processing and responding to threats. Hippocampal volume matured around 4.5 years of age. The hippocampus is involved in many skills, including memory, language and spatial cognition^23^. The improvement of spatial performance observed in children aged 4–5 years could reflect hippocampal maturation^24^. This period also coincides with the offset of infantile amnesia, which has been linked to an immature hippocampus^23^. Prior studies differed on age at which the volumes of different basal ganglia structures peak, from late childhood to adolescence^25,26^. Our results show that basal ganglia volumes (caudate, putamen and pallidum) asymptote around 5 years of age. In our study, thalamus volume was predicted to reach maturation around 7.5 years. The caveat of this observation is that we do not have any observations at that period. Reports on normative developmental trajectory of the thalamus are inconsistent, with peak age varying from 4 to 13 years (refs. ^25,27^). Inconsistencies between our study and previous reports could be due to differences in study design, sample size and population demographics.

Regarding the effects of demographic factors and birth outcomes, sex, adverse birth outcomes and SES influenced ICV and subcortical volumes. ICV was significantly larger in males than in females throughout the age range. This result is consistent with existing literature on sex differences in neuroanatomy, which finds that male brains are larger, on average, than female brains throughout the lifespan^12^. Prior reports on sex differences in subcortical volumes in late childhood through adulthood are inconsistent^12^, perhaps due to differences in study design and cohort age. In our study, all subcortical volumes were larger in males than in females from birth to age 6. This association was robust and remained significant in the replication analysis. However, males had relatively smaller caudate volumes than females when adjusting for ICV. The relative lack of sex differences when adjusting for ICV is unsurprising given collinearity between ICV and individual subcortical volumes and is consistent with prior research (see Methods). The relatively smaller caudate volume in males could relate to sex differences in striatal-associated functions, including motivated behaviors and responsiveness to drugs of abuse, but ought to be considered with caution as the proportions method we used can result in misassignment of structures as larger or smaller than their actual size^28^. We also found that sex plays a significant role in cognitive development, with males having lower visual reception and fine motor scores than females across the age range. There are many factors that could potentially influence sex differences in brain development, such as prenatal and postnatal exposure to gonadal steroids, sex chromosome genes and environmental effects^28^.

Preterm birth and low birthweight were negatively associated with all volumes, but only at intercept. This possibly reflects delayed maturation of regions in early infancy with subsequent catching up in later development. Several studies in children and adults born very preterm with very low birthweight report persistent lower volumes in subcortical structures^14^. However, most children born preterm fall into the category of moderate (32–34 weeks) to late (34–37 weeks) preterm. Worldwide, of all children born preterm, around 84.7% are moderate to late preterm^29^. This group is underrepresented in most studies, and there is a paucity of information on their developmental outcomes. The current study is more representative of these moderate-late preterm children. Associations with ICV, amygdala and hippocampus are robust as evidenced in the replication analysis. During cognitive development, we see significant negative associations with gestational age at birth on all developmental domains tested in MSEL. For visual reception, deficits became more pronounced with age. By contrast, receptive language developed more quickly in preterm children, mirroring our brain volume findings. In general, our results are in keeping with reported deficits in cognitive, language and motor skills in children born preterm^14^.

Maternal education and family income exhibited different patterns of association with our developmental imaging phenotypes, suggesting that parental education and family income represent distinct resources that influence children’s environments and development in different ways^30^. Maternal education could influence the child’s development through both nature and nurture (genes and environment). Higher maternal education is correlated with better health service use, quality of childcare system and mother–child interactions^31^. Maternal education has positive effects on general cognitive ability^32^ and verbal and nonverbal functioning in children^32,33^, patterns also observed in the current study in relation to visual reception and fine motor function.

ICV was significantly lower in children with mothers with primary or secondary education than in children with mothers with tertiary education across the age range. Educational attainment^34^ and ICV^35^ are heritable traits with substantial genetic overlap reported from large-scale genome-wide association studies^34^. These overlapping genetic factors may partially explain the relationship observed here.

At the asymptote, lower maternal education was a predictor for smaller amygdala volume. The amygdala has a critical role in processing threats and learning environmental cues^22^. Early-life stress and risk exposure can impact amygdala volume and function^36–38^. Previous studies, all with sample sizes of less than 1,000, have shown inconsistencies in the direction of associations between parental education and amygdala volume in children^39^. We observe a positive relationship between maternal education and amygdala volume. Our observation is in line with results from a large cohort study of 9,380 children between 9 and 10 years of age^40^. The discrepancies between studies with different sample sizes underscores the need for large datasets in neuroimaging studies to validate associations between imaging phenotypes and different biological and environmental factors. In the sensitivity analysis with only maternal education as the socioeconomic variable, we found lower maternal education to be significantly associated with smaller hippocampal volume. This is consistent with prior reports suggesting a positive relationship between hippocampal volume and parental education^41,42^. Maternal education positively predicted thalamic volume at the asymptote. This finding is consistent with a previous study showing that higher childhood SES, as measured by parental education/occupation, was associated with larger thalamus volume^43^. Anomalous development of the thalamus can significantly affect development of other cortical and subcortical brain structures and can impact cognitive outcomes^44^. At the asymptote, caudate, putamen and pallidum volumes were also significantly lower with lower maternal education, similar to findings observed in previous studies^39^.

Family income can impact many aspects of a child’s environment. Children from lower-income families are more likely to experience stressful environments and possess fewer material and non-material resources, whereas children from higher-income families are reported to have better environmental stimulation that promotes brain development^15^.

Children from lower-income families had significantly smaller hippocampal volumes across the age range. This is consistent with previous reports of associations between SES and hippocampal volume where income was used as a measure of SES^15,39^. The hippocampus mediates long-term memory functions and responses to environmental stress^45^, and larger hippocampal volume is related to better memory performance. Children from high-SES backgrounds have more exposure to stimulating environments that promote learning, including certain educational activities, than children from low-SES backgrounds, and this could improve memory functioning in childhood and boost hippocampal volume^46^.

At the asymptote, thalamic volume was significantly smaller in children from lower-income families, consistent with previous reports^39^. Basal ganglia structures were also smaller, which is similar to that observed in previous studies^47,48^. Early-life stress is known to affect development of the caudate and putamen^49^. Thalamus and basal ganglia networks modulate behavioral responses and regulate cortical neurons. Smaller volume in thalamic and basal ganglia regions in children from lower-SES backgrounds might lead to difficulty in coordinating behavioral responses to stress and reward stimuli^47^. In our study, family income was not a significant predictor of ICV. Previous studies on the effects of SES on ICV yielded conflicting observations, with one study reporting a positive correlation with income–poverty ratio^47^ and one study reporting no association between SES and ICV^50^. Similarly, family income was not significantly associated with amygdala volume, and this observation is consistent with previous studies in individuals from late childhood to adulthood^45^. Lower family income predicted lower scores on visual reception and receptive and expressive language scales of MSEL. This is consistent with prior research linking disparities in SES to several cognitive domains, including overall cognitive development, memory, language acquisition, executive control and school achievement^15^. Effects became more pronounced at later ages, although the only subscale to show a significant interaction effect with age was visual reception.

Prior studies have reported an impact of socioeconomic differences on cognitive and brain development as early as the first year^15^. Our observations provide evidence of differential effects of SES factors on brain development as early as birth. However, it must be noted that split-sample replication analyses suggest that brain volume associations with SES are less robust than associations with sex and adverse birth outcomes. The subtle effect of these factors on brain structure may be due to genetic, environmental and gene × environment interaction effects on neurogenesis, synaptogenesis and neuronal morphology^11^.

Regarding question 3 (‘what are the neural correlates of cognitive development in this period?’), in children born full term, globus pallidus and caudate volumes were positively correlated with visual reception scores. These associations make sense as the primary abilities assessed by visual reception scale are visual discrimination, memory, organization, sequencing and spatial awareness^51^. Basal ganglia structures are known to interact with the prefrontal cortex to support working memory. Furthermore, the caudate and globus pallidus facilitate visual motor integration via corticostriatal loops^52^. In children born preterm, hippocampal volume was positively correlated with visual reception scores, although sensitivity and split-sample replication analyses suggest that this effect may not be robust. The hippocampus plays a significant role in cognition and memory, particularly sequencing^23,53^, and lower hippocampal volume has been linked to worse cognitive outcomes in children born preterm^14^. ICV was negatively correlated with gross and fine motor scores in children born full term. This relationship may be due to changes in gray matter and white matter composition, myelination or cerebral spinal fluid in the subarachnoid space^54–56^.

Mediation analyses suggest that the effects of sociodemographic factors on cognitive scores are partially mediated by brain volumes with significant but small effect sizes. The effects of low maternal education and low family income on visual reception scores are partially mediated by lower caudate and pallidum volumes, respectively. Similar to what we observe, the pallidum has been found to partially mediate the relationship between diminished growth and intelligence quotient in children from low-SES conditions^57^. In addition, the association between male sex and lower visual reception is partially ameliorated by caudate and globus pallidus volumes. In children born preterm, the effect of male sex on visual reception score is ameliorated by larger hippocampal volume, and the effect of lower maternal education is partially mediated by lower hippocampal volume.

A particular strength of our study is the large ethnically and socially diverse sample of children who have undergone cognitive assessments and structural MRI measurements in the critical period of infancy and early childhood. Because our study is well powered and we apply stringent corrections for multiple comparisons, results are expected to be rigorous and robust; however, there are some limitations. First, we used maternal education as a binary variable (tertiary versus primary/secondary education). However, in the South African cohort, most of the mothers had some secondary education, and very few had tertiary education. This is an accurate reflection of the country, where 79% of women have an upper secondary education as the highest level achieved, but only 6% of women have a tertiary education^58^. The binary classification might underrepresent some characteristics of that population. Second, as with other multisite observational studies, uncontrolled confounding may influence results. For putamen and pallidum volumes at birth, the group with low maternal education is predominantly from the South African cohort, which is mainly composed of people of African descent and mixed ancestry. They have larger volumes in the pallidum and putamen than people of European descent in this study in the same age range. Hence, the observed effect of maternal education on the putamen and pallidum at birth could reflect differences in segmentation protocols, health history of parents and ancestry within that cohort. Third, although we have an adequate number of observations across the age range to draw representative inferences, we also note that the number of observations is higher from birth to age 3 years. The current results along with others strongly point to the need of inclusion of different ethnic populations from diverse socioeconomic strata in neuroimaging studies. The ORIGINs consortium is a step toward achieving this goal.

In conclusion, our study contributes to the better understanding of the effects of sex, adverse birth outcomes and SES on neurodevelopment and cognitive outcomes in socially and ethnically diverse cohorts. Our approach could be expanded to address other environmental and contextual factors that shape early brain development and, in the long term, inform public health policy and interventions. Furthermore, by defining trajectories of ICV and subcortical volume development in infancy and early childhood, we lay the foundation for large-scale imaging–genetics studies of this critical developmental period.

## Methods

### Participants

The data for this project were provided by the following members of ENIGMA-ORIGINs: Max Planck Institute for Human Cognitive and Brain Sciences (Germany), GUSTO (Singapore), the Drakenstein Child Health Study (South Africa), the BCP, Boston Children’s Hospital/Harvard Medical School, the IBIS network, University of North Carolina EBDS and UCI. The final dataset includes children representing socially and ethnically diverse backgrounds (Table 1 and Supplementary Table 17). Each project was approved by their respective local review board, and informed consent was obtained from parents/legal guardians and children before data collection. The reviewing organizations include Michigan State University; Max Planck Institute for Human Cognitive and Brain Sciences, Germany; National University of Singapore, Singapore; University of Cape Town, South Africa; the University of North Carolina, Chapel Hill; the University of California, Irvine; and Boston’s Children Hospital. For three cohorts (Germany, South Africa and UCI) MRI data were cross-sectional. The other five cohorts had longitudinal data. Overall, the imaging cohort included 2,108 children with a total of 3,607 observations and an age range of 5–2,250 postnatal days (Supplementary Fig. 1). No statistical methods were used to predetermine sample sizes, but our sample sizes are among the highest for pediatric imaging studies. As this was an observational study, blinding does not apply.

### Image acquisition and analysis

Structural T1-weighted and T2-weighted scans of each participant were acquired and processed at each study site (Supplementary Tables 18–20). Images were acquired at different field strengths (1.5 T and 3 T). The reported sample size from each cohort is after quality control was performed locally at the respective sites.

### Cohort characteristics

#### Max Planck Institute for Human Cognitive and Brain Sciences

The Max Planck Institute for Human Cognitive and Brain Sciences cohort includes children with and without a family risk of dyslexia who underwent MRI between 3 and 6 years of age. Exclusion criteria included impaired hearing and/or vision, an intelligence quotient below 80, psychiatric disorders, attention-deficit/hyperactivity disorder, previous neurosurgery, contraindication for MRI, medication that modulates brain function and inability and/or unwillingness to follow experimental instructions and/or perform experimental tasks. Participating families received travel cost reimbursement and a small gift.

##### Segmentation protocol

Scans used for segmentation were preselected for image quality by visual inspection for artifacts, signal dropouts, spatial distortion and anatomical anomalies. In the sample of 3- to 6-year-old children, segmentation was performed using the recon-all procedure implemented into FreeSurfer, which allowed for the extraction of gray matter images from the T1-weighted scans. First, 130 images were skull stripped. Second, white matter and gray matter boundaries were reconstructed. Third, boundary reconstructions were used to calculate the pial surface. These automatic processing steps could not be completed in three datasets, which were then discarded so that cortical surface reconstructions were available for 127 individuals. ICV was calculated using an atlas-based estimation approach implemented in FreeSurfer (https://surfer.nmr.mgh.harvard.edu/fswiki/eTIV). Specifically, they computed a volume-scaling factor derived by spatially transforming each individual image to an atlas image for which the ICV is already known. This volume-scaling factor renders a reliable estimation possible because it is highly correlated with the individual ICV. Quality of the automatic surface reconstruction results was assessed by thorough visual inspection. To ensure the neuroanatomical accuracy of each individual dataset, remaining parts of the skull were removed, and removed parts of the cortex were added again by adding control points and rerunning the surface reconstruction if necessary.

#### GUSTO

This study is comprised of a parent–offspring cohort. Exclusion criteria included mothers receiving chemotherapy or psychotropic drugs or type I diabetes mellitus. Participant compensation for the families was SGD $100 per trip.

##### Segmentation protocol

For neonates, a Markov random field model was used to automatically segment the subcortical structures. In the Markov random field model, the prior probability of each structure was computed based on the manual segmentation of 20 participants randomly chosen from the participants with the manual labels. The prior probability atlas was obtained in the GUSTO neonatal atlas^59^ where all T2-weighted images were nonlinearly transformed to using large deformation diffeomorphic metric image mapping^60^. Accuracy of this automated segmentation was validated using leave-one-out validation in the manual segmented dataset. ICV was calculated as the number of voxels inside the brain after brain skull removal and scaled by the image resolution, including gray matter, white matter and cerebrospinal fluid of ventricles^61–63^.

For older children, to eliminate potential profound effects of head motion on our statistical results, we manually checked image quality based on the stringent criteria in Ducharme et al.^64^. Disqualified images were excluded from this study. FreeSurfer software (https://ddec1-0-en-ctp.trendmicro.com:443/wis/clicktime/v1/query?url=http%3a%2f%2fsurfer.nmr.mgh.harvard.edu%2f&umid=eb095c3f8b31464688adef826b8ef738&auth=8d3ccd473d52f326e51c0f75cb32c9541898e5d5-e74d695f31fbfaabebae9b32a93056ff6e20c8e6) was then used to label each voxel in the usable T1-weighted image as gray matter, white matter, cerebrospinal fluid or subcortical structures. FreeSurfer used a Markov random field model that requests a prior probability obtained from a training dataset with T1-weighted images and their manual structural labels. We reconstructed the prior probability in the Markov random field model based on the manual segmentation of 30 children and embedded it in FreeSurfer. A postprocessing quality check was conducted following the instructions in https://ddec1-0-en-ctp.trendmicro.com:443/wis/clicktime/v1/query?url=https%3a%2f%2fsurfer.nmr.mgh.harvard.edu%2ffswiki%2fFsTutorial%2fTroubleshootingData&umid=eb095c3f8b31464688adef826b8ef738&auth=8d3ccd473d52f326e51c0f75cb32c9541898e5d55e1ff822a43b798d1a48a3fb6506b120f651b211. Segmentation accuracy was assessed using a volume overlap ratio between the automated and manual segmentations.

#### Drakenstein Child Health Study, University of Cape Town

This longitudinal cohort aims to investigate the determinants of child growth, health and development in a stable, semiurban, low-socioeconomic-status community in South Africa. For the current study, children with scans acquired in the first month after birth are included. Exclusion criteria were minimal to maximize generalizability and were focused primarily on individuals who did not live in the region and thus could not be readily followed-up or who intended to move out of the district within the following 2 years. Participants were compensated for their time and travel expenses at each study visit with a voucher/gift card to the value of 350 ZAR (South African Rand). Refreshments were made available during the visit. Travel arrangements were offered to those participants who resided outside the study area.

##### Segmentation protocol

Sagittal three-dimensional T2-weighted images from 2- to 6-week-old infants were brain extracted with FSL v5.0. The output images were preprocessed further in Statistical Parametric Mapping software (SPM8) run in MATLAB R2017B. Images were registered and normalized with modulation to the University of North Carolina neonate T2 template^65^. Hereafter, images were segmented into gray matter, white matter and cerebrospinal fluid based on the corresponding neonate probabilistic maps. Gray matter segmentations from 140 infants passed quality control through visual inspection (exclusion low image quality: 18 images; exclusion poor segmentation: 17 images). Gray matter volumes were extracted according to the automated anatomical labeling atlas^66^, adapted for neonates^65^, for the left and right amygdala, hippocampus, thalamus, caudate, putamen and pallidum.

#### University of North Carolina EBDS

This prospective longitudinal cohort includes children at high familial risk for schizophrenia and bipolar illness, a ‘structural’ high-risk group (children with prenatal isolated mild ventriculomegaly), a large sample of twins and an exceptionally large sample of typically developing infants. Exclusion criteria at enrollment included major medical illness in the mother, abnormality on ultrasound and current substance abuse. For participation in the study, parents received US $50 per child for each MRI visit and US $50 per child for each developmental assessment visit.

##### Segmentation protocol

For neonates, hippocampus and amygdala segmentation was performed using a multimodality, multitemplate-based automatic method combining T1- and T2-weighted high-resolution images in AutoSeg v3.3.2 (ref. ^67^) using the same multitemplate library as in the UCI cohort. Other subcortical structures were determined via a multimodality, single-template-based automatic method combining T1- and T2-weighted high-resolution images in AutoSeg v3.3.2 using the same single template as in the UCI cohort. For participants older than neonate age, all subcortical structure segmentation was performed using a multimodality, multitemplate-based automatic method combining T1- and T2-weighted high-resolution images in MultiSeg Pipeline v 2.2.1 using the same templates as in the IBIS cohort.

#### IBIS network

This longitudinal study aims to examine the early brain and behavioral development in infants at familial risk for autism and low-risk control infants (LR). For the current study, participants enrolled in the LR group were included. Exclusion criteria included the following: (1) diagnosis or physical signs strongly suggestive of a genetic condition or syndrome (for example, fragile X syndrome) reported to be associated with autism spectrum disorders, (2) a significant medical or neurological condition affecting growth, development or cognition (for example, CNS infection, seizure disorder and congenital heart disease), (3) sensory impairment, such as vision or hearing loss, (4) low birthweight (<2,000 g) or prematurity (<34 weeks gestation), (5) possible perinatal brain injury from exposure to in utero exogenous compounds reported to likely affect the brain adversely in at least some individuals (for example, alcohol and selected prescription medications), (6) non-English-speaking families, (7) contraindication for MRI (for example, metal implants), (8) individuals who were adopted and (9) a family history of intellectual disability, psychosis, schizophrenia or bipolar disorder in a first-degree relative. In addition, LR infants were excluded for autism spectrum disorder based on clinical evaluation at 24 and/or 36–60 months of age. All IBIS families were reimbursed for expenses incurred during study participation (for example, travel, lodging and meals). Families also received compensation for each of the longitudinal study visits, and children were offered small toys for participating.

##### Segmentation protocol

All subcortical structure segmentation was performed using a multimodality, multitemplate-based automatic method combining T1- and T2-weighted high-resolution images in AutoSeg v3.3.2 (ref. ^67^), followed by manual correction of selected datasets in ITK-Snap^68^, if necessary. The multitemplate datasets consisted of 16 6-month-old datasets for the 6-month-old participant processing as well as 16 1-year-old and 16 2-year-old datasets for the 1- to 2-year-old participant processing.

#### UCI

This is a prospective, longitudinal, follow-up study in a population-based cohort. For the current study, infants with MRI data in the first 2 months after birth were included. Exclusion criteria included (1) preterm birth <34 completed weeks gestation, (2) maternal use of psychotropic medication during pregnancy, (3) maternal use of corticosteroids during pregnancy, (4) maternal smoking and drug use during pregnancy (self-reports verified by urinary cotinine and drug toxicology), (5) congenital or genetic disorder (for example, fetal alcohol syndrome, Down syndrome and fragile X) and (6) major neurologic disorder at birth (for example, bacterial meningitis and epilepsy). Participant compensation was US $100 per scan.

##### Segmentation protocol

Hippocampus and amygdala segmentation was performed using a multimodality, multitemplate-based automatic method combining T1- and T2-weighted high-resolution images in AutoSeg v3.3.2 (ref. ^67^), followed by manual correction of all datasets in ITK-Snap^68^. Images were manually corrected in both original and left–right mirrored presentation to account for asymmetric presentation biases^69^, and volumes were averaged for the two presentations. The multitemplate datasets consisted of eight neonate participants. Other subcortical structures were determined via a multimodality, single-template-based automatic method combining T1- and T2-weighted high-resolution images in AutoSeg v3.3.2. The single template was a single, unbiased average atlas computed from the ALBERT^70^ datasets.

#### BCP

This sequential cohort with an accelerated longitudinal study design included typically developing children between birth and 5 years of age recruited across two data collection sites (University of North Carolina and The University of Minnesota). Exclusion criteria included gestational age of <37 weeks, birthweight of <2,500 g and any major pregnancy and/or delivery complications. Participation compensation was US $150 Target gift card or Visa card (US $135 + US $15 for travel equivalent reimbursement) for each completed visit (scan and assessments). Participants who completed an MRI retry scan (without an assessment) were given US $75 (Target or Visa card).

##### Segmentation protocol

All subcortical structure segmentations were performed using a multimodality, multitemplate-based automatic method combining T1- and T2-weighted high-resolution images in the MultiSeg Pipeline v 2.2.1 without manual correction. The multitemplate datasets consisted of 16 6-month-old datasets for participants younger than 9 months of age as well as 16 1-year- and 16 2-year-old datasets for participants older than 9 months of age using the same templates as in the IBIS cohort.

#### Boston Children’s Hospital/Harvard Medical School

This is a prospective, longitudinal cohort aimed to study neural development in children with and without a familial history of developmental dyslexia. Exclusion criteria include psychiatric or neurological illness, sensory impairment, contraindications for MRI studies (for example, magnetic resonance-incompatible metal implants, such as surgical clips, and probability of metal fragments embedded in the body), treatment with psychotropic medication, prematurity and an atypical hearing screening. Each family received a US $50 gift certificate for a local bookstore for their participation for each MRI session per participant (infant/child and parent). Families received an additional US $25 per session for parking (US $10), transportation costs (US $10) and small toys/prizes (US $5).

##### Segmentation protocol

All images were processed using (1) infant FreeSurfer^71^ for scans that were taken between 0 and 3 years of life and (2) a modified FreeSurfer pipeline adjusted for processing MRI data from children acquired at age 4.5 years or older. Infant FreeSurfer is an automated segmentation and surface extraction pipeline designed to accommodate clinical MRI studies of infant brains in a population of 0- to 2-year-old children. The algorithm relies on a single channel of T1-weighted MRI images to achieve automated segmentation of cortical and subcortical brain areas, producing volumes of subcortical structures and surface models of the cerebral cortex. Infant FreeSurfer is equipped with niftyreg (https://sourceforge.net/p/niftyreg) for automated nonlinear registration between template and individual brains. The standard FreeSurfer pipeline^72^ has been optimized to perform well on adult acquisitions; however, it has been shown that with expert guidance and good-quality data, the tools can be used on images of participants as young as 4.5 years of age^73^. ICV was calculated by the approach mentioned in infant FreeSurfer (see Max Planck Institute for Human Cognitive and Brain Sciences) but recomputed for infants using templates/images from the Developmental Human Connectome Project. After inspection of the segmentation and surface reconstruction outcomes, 85 of the youngest scans were processed with a variation of infant FreeSurfer. Instead of relying on the default multiatlas label-fusion segmentation framework, they used the newly released sequence-adaptive whole-brain segmentation^74^ framework with an infant atlas for volumetric segmentation, improving their accuracy.

### Cognitive assessment

Cognitive functioning was measured using the MSEL^51^; this assessment has a high test–retest reliability. The battery consists of 144 items that are distributed across five main subtests: expressive and receptive language, visual reception and fine and gross motor function. Raw scores can be used to generate standardized norm-referenced *T* scores, percentile ranks and age-equivalent scores. We chose to focus on raw scores, as we were interested in actual changes in children’s abilities over time rather than their degree of difference from a normative sample^75^. Raw scores for gross motor scales were available within the range of 75 to 1,275 d. Fine motor scale and visual reception scale data were available within the range of 75 to 1,776 d. Expressive and receptive language scores were available within the range of 75 to 2,963 d. The demographic distribution of children (*N* = 1,238; observations = 2,530) in the cognitive development analysis is provided in Supplementary Table 21.

### Predictive measures

Birth measures were obtained from hospital records. A gestational age of <259 d was considered preterm, and a birthweight of <2,500 g was considered low birthweight. Parent-reported measures of their educational attainment and income were used to assess socioeconomic status. Maternal education was categorized as primary, secondary and tertiary based on The International Standard Classification of Education. Primary and secondary education were classified as low maternal education. The low-income variable was defined per country-specific norms. For Singapore, low income was <SGD $2,000 per month^76,77^; for South Africa, low income was <1,000 Rand per month^78^; for the United States, low income was <US $50,000 per year^79^. Income was not collected in the German sample (Max Planck). Consequently, this sample is not included in the main analysis but is included in the first sensitivity analysis.

### Statistical analysis

To analyze the age-related growth of ICV and subcortical structures (*l* = 1,…, *L*), we used nonlinear mixed models^80^ with the following asymptotic function:

1 $${f}_{l}\left({x}_{{ij}},{{\boldsymbol{\theta }}}_{{jk}}\right)={\theta }_{{jk}1}+\left({\theta }_{{jk}2}-{\theta }_{{jk}1}\right){e}^{-{e}^{{\theta }_{3}{x}_{{ij}}}}$$

where *x*_*ij*_ is the age of the *i*th observation for the *j*th participant, ***θ***_*jk*_ is a vector of participant- and covariate-specific parameters defining the function, where *θ*_*jk*1_ is the asymptote, *θ*_*jk*2_ is the intercept, and *θ*_3_ is the rate constant that is proportional to the relative rate of increase. This last parameter is not indexed by participant or covariate because it is fixed in the model, whereas the asymptote and intercept had both fixed and random effects as

2 $${\theta }_{{jk}1}={\theta }_{1}+\mathop{\sum }\limits_{k=1}^{K}{\beta }_{k1}+{u}_{j1}$$

where ${\theta }_{1}$ is a fixed population parameter, the fixed effect *β*_*k*1_ is covariate specific (*k* = 1,…, *K*), and the random effect ${u}_{j1}$ is participant specific (*j* = 1,…, *J*). Likewise, *θ*_*jk*2_ follows Eq. (2). The fixed effects were preterm birth, sex, low birthweight, low maternal education, low family income and cohort. All fixed effects were coded as binary effects using dummy variables. The simplified form of the nonlinear mixed model is

3 $${y}_{{ijl}}={f}_{l}\left({x}_{{ij}},{{\boldsymbol{\theta }}}_{{jk}}\right)+{\varepsilon }_{{ijl}}$$

where *y*_*ijl*_ is the ${ij}$ th observation for the *l*th subcortical structure, and *ε*_*ijl*_ is an error term. The model error was assumed to follow a normal distribution with heterogenous variances between cohorts, but this was not formally tested.

Multiple-comparisons correction for 35 tests (7 volumes and 5 covariates) was applied by using Bonferroni correction with an original *α* level set at 0.05, resulting in a *P* value significance threshold of *P* = 0.001. For the main analysis and first sensitivity analysis, we used raw volumes, which are advantageous for comparisons across studies and for creating normative volumetric values for the age range^81^. Prior studies have shown that ICV correction can influence the interpretability of brain–behavior associations across ages. For example, Dhamala et al. reported that ICV correction reduces predictive accuracies for cognitive ability from gray matter volumes^82^. Moreover, ICV correction methods reduce both univariate sex differences and the accuracy of multivariate sex prediction based on gray matter volume^82–84^. Finally, ICV and different brain regional volumes have unique growth trajectories across development such that correction for ICV will have different effects across the different periods^81,85^.

For the second sensitivity analysis, the volumes were ICV scaled and modeled with a linear mixed model with covariate- and cohort-specific fixed effects, participant-specific random effects and cohort-specific error variances. The linear mixed model used for this sensitivity analysis is

4 $${y}_{{ijl}}/{{ICV}}_{{ijl}}* 1000={\mu }_{l}+\mathop{\sum }\limits_{k=1}^{K}{\beta }_{{kl}}+{u}_{{jl}}+{\varepsilon }_{{ijl}}$$

where ${\mu }_{l}$ is the overall mean for the *l*th subcortical structure, *β*_*kl*_ is covariate-specific and cohort-specific fixed effects, and *u*_*jl*_ is the *j*th participant random effect. *ε*_*ijl*_ has the same specifications as before.

To investigate if genetic relatedness affects the results of the analysis, we performed a third set of sensitivity analyses, removing a single twin/sibling from the EBDS cohort and rerunning the main model.

Cognitive changes across age were modeled with a linear mixed model for each cognitive scale. The linear mixed model used on the evaluation of cognitive changes across age is

5 $${y}_{{ijm}}={\mu }_{m}+{x}_{{ijm}}+\mathop{\sum }\limits_{k=1}^{K}{\beta }_{{km}}+{u}_{{jm}}+{\varepsilon }_{{ijm}}$$

where *y*_*ijm*_ is the *i*th observation on the *j*th participant for the *m*th cognitive score, *μ*_*m*_ is the overall mean, *x*_*ijm*_ is the slope given by age, $\mathop{\sum }\nolimits_{k=1}^{K}{\beta }_{{km}}$ is the sum of the covariate and cohort fixed effects, ${u}_{{jm}}$ is the *j*th participant’s random effect, and *ε*_*ijm*_ is the error term for the *m*th model. Both random effects and the error term follow a normal distribution, where the random effects are assumed independent, and the error term has cohort-specific variances (heterogenous variances). The analysis was performed using the lme4 package^86^ v1.1.31 in R. Multiple-comparisons correction for 25 tests (5 cognitive scores and 5 covariates) was applied by using a Bonferroni correction with an original *α* level set at 0.05, resulting in a *P* value significance threshold of *P* = 0.002.

To analyze the relationship between ICV and subcortical volumes and cognitive scores, Pearson’s correlation was used. The predicted volumes at age 2 were tested for correlation with predicted cognitive scores at age 2 separately in children born preterm and full term. The brain volumes and cognitive measures were not acquired contemporaneously. We focused on age 2 for this analysis as it had dense data points, and gross motor skills is used only until 33 months. Furthermore, cognitive ability at age 2 is a strong predictor of cognitive outcomes at school age^19,87^. A multiple-comparisons correction for 35 tests (5 cognitive scores and 7 brain volume measures) was applied by using a Bonferroni correction with an original *α* level set at 0.05, resulting in a *P* value significance threshold of *P* = 0.001. We also performed a bootstrap method (bootcorci package v 0.0.0.9 in R^88^) to compute confidence intervals for the differences in correlation coefficients between the full-term and preterm groups (Supplementary Table 19).

We additionally performed a replication analysis to test the robustness of the results. The whole sample was randomly split into two folds, and we replicated the analysis (volume trajectory, development of cognitive and motor scores and correlation analysis) 100 times. We have reported the proportion of times both the folds showed the same direction of effect and proportion of times where the results from both folds were of the same sign and significant, which is a more stringent approach.

To test if brain volumes mediate relationships between predictors and cognition, we examined the indirect effects wherever significant brain volume–cognitive score correlations were observed and for those predictors (sex, birthweight, maternal education and family income) that were associated with volumetric measures and cognitive scores. The effects were reported as 95% confidence intervals (significant when they did not include 0) based on 10,000 bootstrapped samples using the mediation package V.4.5.0 in R^89^.

#### Addressing site-dependent variability

Data generated from different cohorts may be subject to systematic differences due to the technologies used to collect and process imaging data as well as systematic differences due to biological effects not fully accounted for in the model (for example, geographical differences). Therefore, our models contemplated the possibility that cohorts may have systematic differences in the mean and the scale of the traits. Specifically, all of our models included the random effect of the cohort on the outcomes (this adjusts for mean differences between cohorts) as well as cohort-specific error variances, which account for possible scale differences. Furthermore, we checked the distribution of model residuals (Supplementary Figs. 9 and 10). The residual plots show that before modeling the distribution, the volumes are clearly bimodal. This primarily reflects differences in age both within and between cohorts but may also reflect effects due to birth outcomes, sociodemographic factors and cohorts. The histogram of residuals shows that after modeling, the residuals are reasonably normal, suggesting that the model accounted for differences due to the effects included in it, including cohort. For thalamus, amygdala, putamen and pallidum volume, we do observe that the Cape Town and UCI cohorts deviate slightly from the normal distribution. We performed another sensitivity analysis removing these cohorts, and the results reaffirm that our inferences are robust (Supplementary Table 22).

### Reporting summary

Further information on research design is available in the Nature Portfolio Reporting Summary linked to this article.

## Online content

Any methods, additional references, Nature Portfolio reporting summaries, source data, extended data, supplementary information, acknowledgements, peer review information; details of author contributions and competing interests; and statements of data and code availability are available at 10.1038/s41593-023-01501-6.

## Supplementary information

Supplementary Information Supplementary Figs. 1–11 and a list of ORIGINs consortium members contributing to this study.

Reporting Summary

Supplementary Tables 1–22 Supplementary Tables 1–22 as a workbook with multiple tabs.

## Figures and Tables

**Extended Data Table 1:**
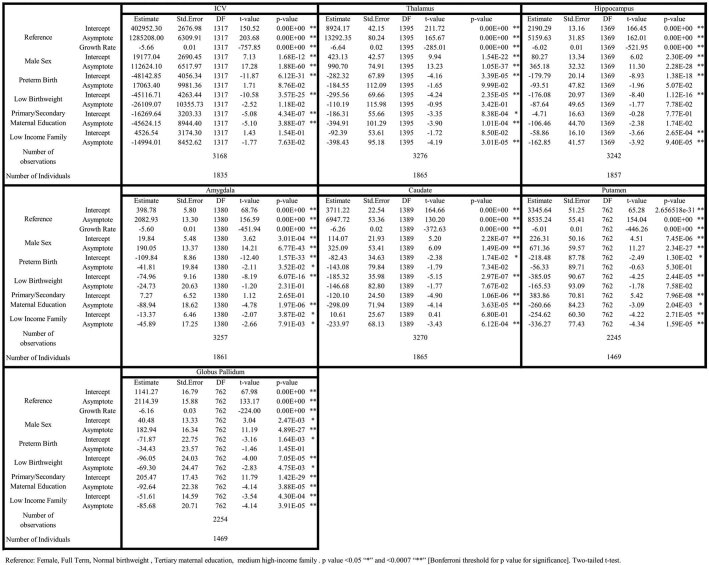
Birth outcomes and socio-demographic factors have significant effects on structural brain development

**Extended Data Table 2:**
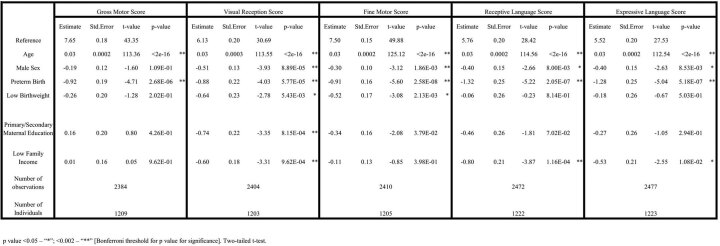
Demographic, birth outcomes, and socioeconomic factors significantly influence cognitive and motor development

## Data Availability

The data for the study came from eight different cohorts. The data for four of the cohorts are deposited in the National Institute of Mental Health Data Archive (NDA) and can be accessed by submitting a data access request to the NDA. Imaging data for twins in the EBDS cohort are available through NDA 1974 and NDA 2384 and for singletons via NDA 4314. Imaging data from IBIS are available via NDA 19 and NDA 2027. Imaging data for UCI are available via NDA 1890. Imaging data for BCP are available via NDA 2848. Imaging data from the Harvard cohort and a subset of EBDS, IBIS and BCP data will also be made available through NDA 3905. Cognitive data from all cohorts and imaging data for the other cohorts may be available upon request to the parent cohort and may require Institutional Review Board approval or data use agreements. Investigators interested in further information on ORIGINs dataset access and sharing can contact the corresponding author.

## Code availability

All code used in the analysis in this paper is available in the GitHub repository at https://github.com/knickmeyer-lab/ORIGINs_ICV-and-Subcortical-volume-development-in-early-childhood.
