# Supplementary material for: A global multicohort study to map subcortical brain development and cognition in infancy and early childhood
Source: Nat Neurosci. 2023 Nov 23;27(1):176–86. doi: 10.1038/s41593-023-01501-6 (PMC10774128; doi:10.1038/s41593-023-01501-6)
Supplement: Supplementary file 1 — Supplementary Figs. 1–11 and a list of ORIGINs consortium members contributing to this study. [file 41593_2023_1501_MOESM1_ESM.pdf]

nature neuroscience

Resource

https://doi.org/10.1038/s41593-023-01501-6

A global multicohort study to map subcortical brain development and cognition in infancy and early childhood

In the format provided by the authors and unedited

Supplementary Figures

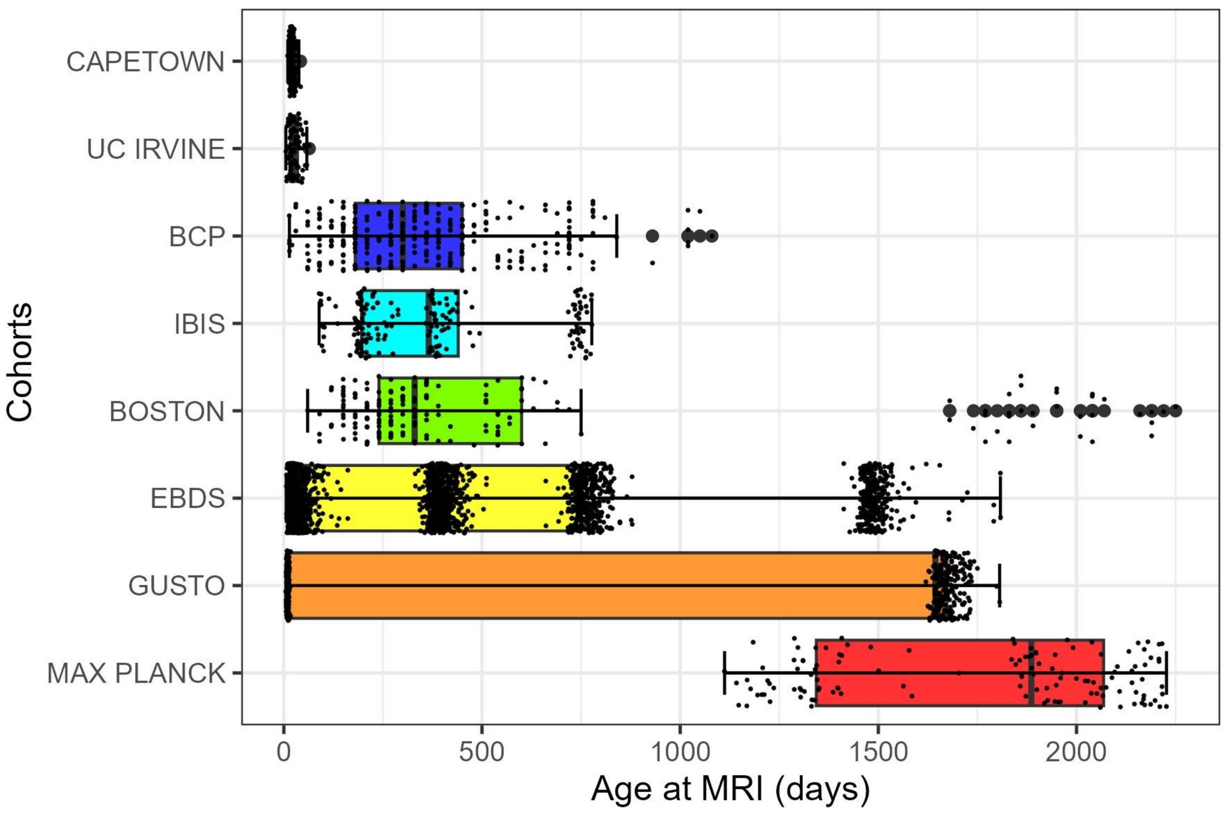

Figure 1: Age distribution of participants in the different cohorts. In the box, the lower boundary is the first quartile(Q1), the upper boundary is the third quartile (Q3), and the line represents the middle (50%) of the data. The bounds of whiskers represent the range of the data. Number of individuals / Number of observations for CAPETOWN – 135/135, UC IRVINE – 88/88, BCP -172/317, IBIS – 86/186, BOSTON -130/181, EBDS – 1013/2152, GUSTO – 357/421, MAX PLANCK – 127/127. Summary stats for CAPETOWN, age range is 10-42 days, median 21 days, quartiles (q1- 18 and q3 - 26), and 90th percentile 31; UC IRVINE age range is 5-64 days, median 24 days, quartiles (q1- 17 and q3 - 35), and 90th percentile 45; BCP, age range is 14-1080 days, median 300 days, quartiles (q1- 180 and q3 - 450), and 90th percentile 720; IBIS age range is 89-777 days, median 366 days, quartiles (q1- 196 and q3 - 440), and 90th percentile 183; BOSTON, age range is 60-2250 days, median 330 days, quartiles (q1-240 and q3 - 600), and 90th percentile 1860; EBDS, age range is 6-1808 days, median 380 days, quartiles (q1- 30 and q3 - 751), and 90th percentile 1475;GUSTO, age range is 5-1806 days , median 1644 days, quartiles (q1- 10 and q3 - 1673), and 90th percentile 1701;MAX PLANCK, age range is 1112-2227 days , median 1886 days, quartiles (q1- 1344 and q3 - 2068), and 90th percentile 2172.

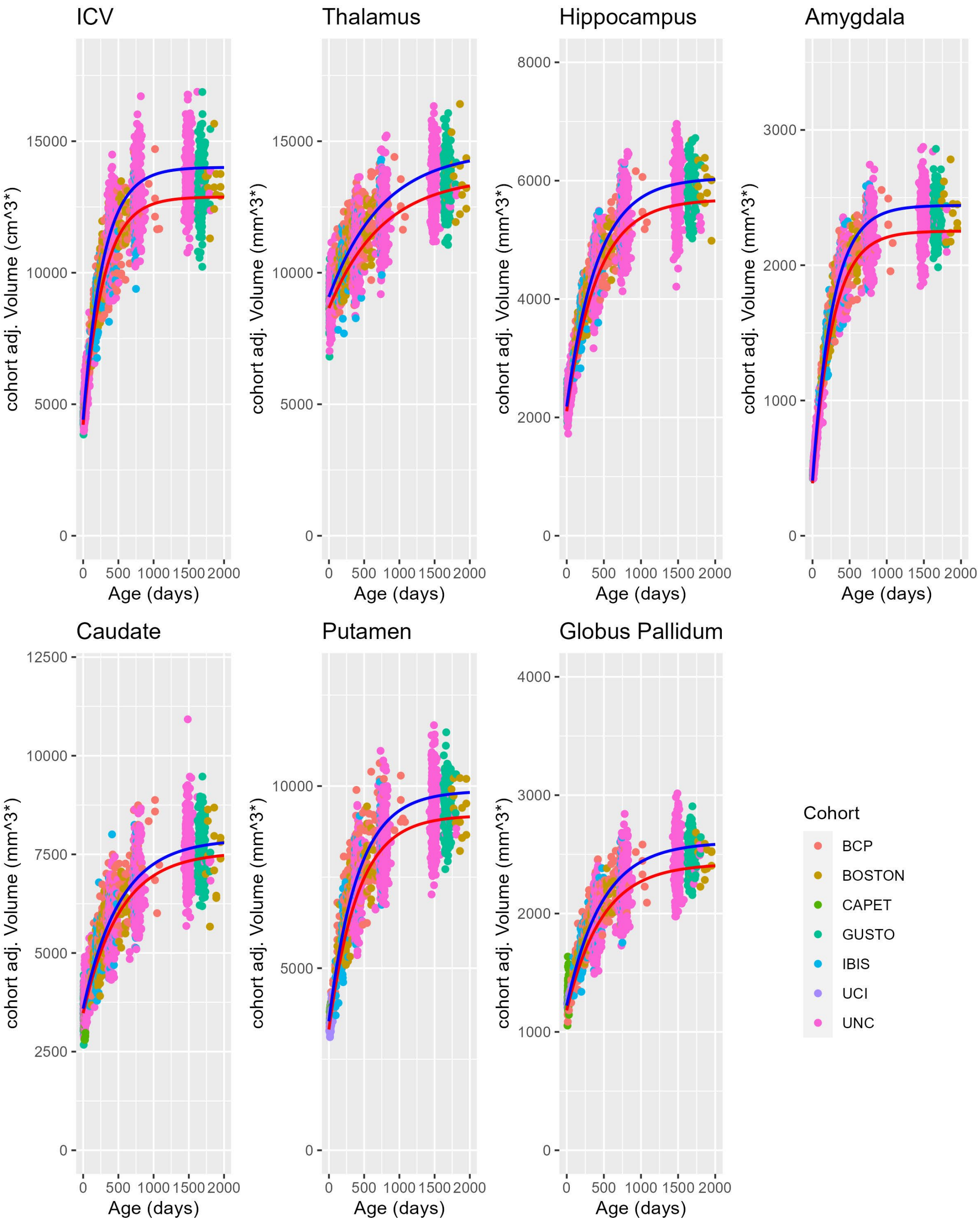

Supplementary Figure 2: Effect of sex on developmental trajectories of ICV and subcortical brain structures. Trajectory of males is represented by blue line and that of females by red line. Individual data points are shown in the circles and colors represent the cohorts. ICV- Intracranial Volume. BCP: Baby Connectome Project; BOSTON: Boston's Children Hospital (BCH)/Harvard Medical School; CAPET: Drakenstein Child Health Study, Cape Town; GUSTO: Growing Up in Singapore Towards healthy Outcomes, Singapore; IBIS: Infant Brain Imaging Study Network; UCI: University of California Irvine; UNC: UNC Early Brain Development Study

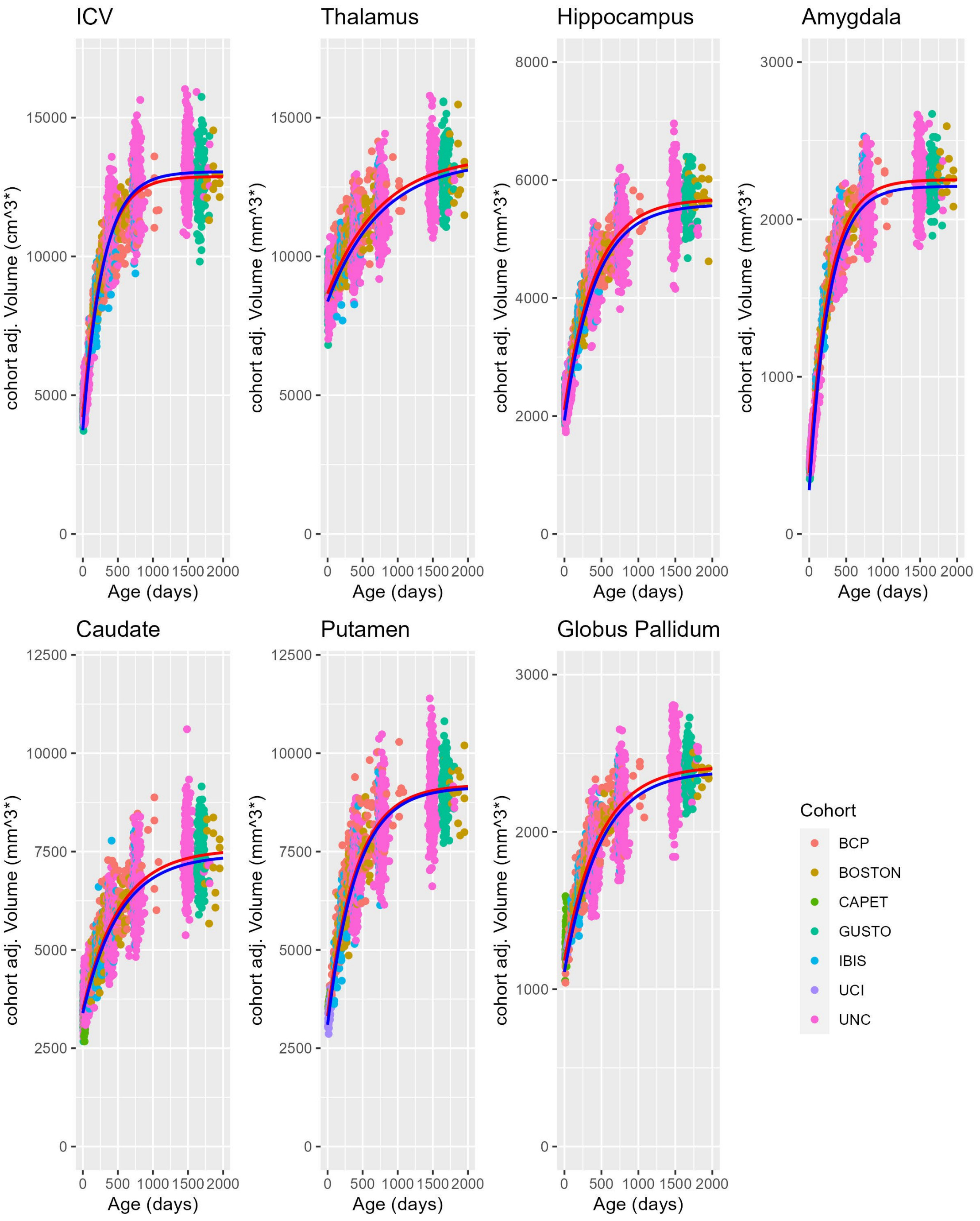

Supplementary Figure 3: Effect of preterm birth on developmental trajectories of ICV and subcortical brain structures. The trajectory of preterm born children is represented by blue line and that of full term born children by red line. Individual data points are shown in the circles and colors represent the cohorts. ICV- Intracranial Volume. BCP: Baby Connectome Project; BOSTON: Boston's Children Hospital (BCH)/Harvard Medical School; CAPET: Drakenstein Child Health Study, Cape Town; GUSTO: Growing Up in Singapore Towards healthy Outcomes, Singapore; IBIS: Infant Brain Imaging Study Network; UCI: University of California Irvine; UNC: UNC Early Brain Development Study

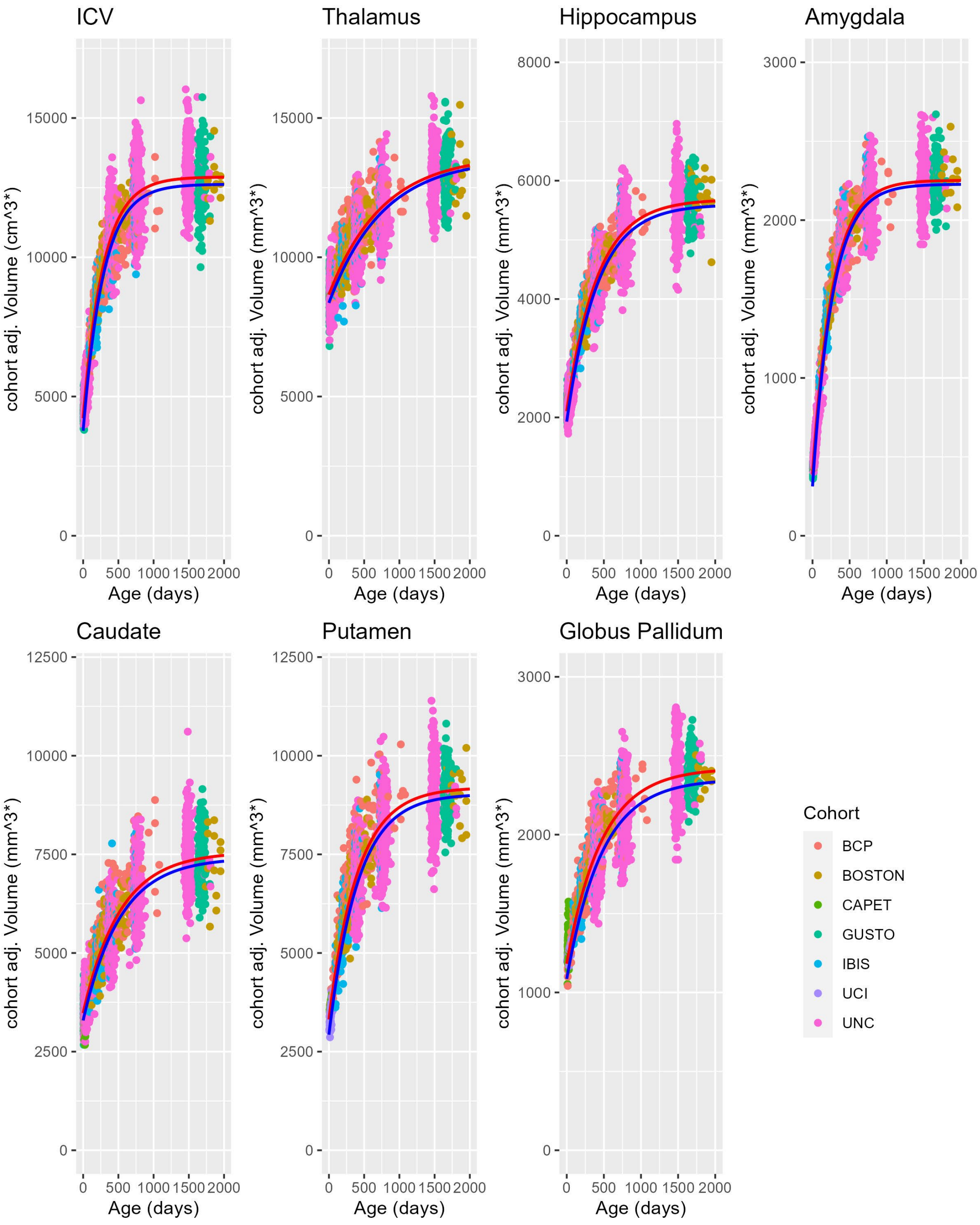

Supplementary Figure 4: Effect of low birthweight on developmental trajectories of ICV and subcortical brain structures. Trajectory of children born with low birthweight is represented by blue line and that of children with normal birthweight by red line. Individual data points are shown in the circles and colors represent the cohorts. ICV- Intracranial Volume. BCP: Baby Connectome Project ;BOSTON: Boston's Children Hospital (BCH)/Harvard Medical School; CAPET: Drakenstein Child Health Study, Cape Town; GUSTO: Growing Up in Singapore Towards healthy Outcomes, Singapore; IBIS: Infant Brain Imaging Study Network; UCI: University of California Irvine; UNC: UNC Early Brain Development Study

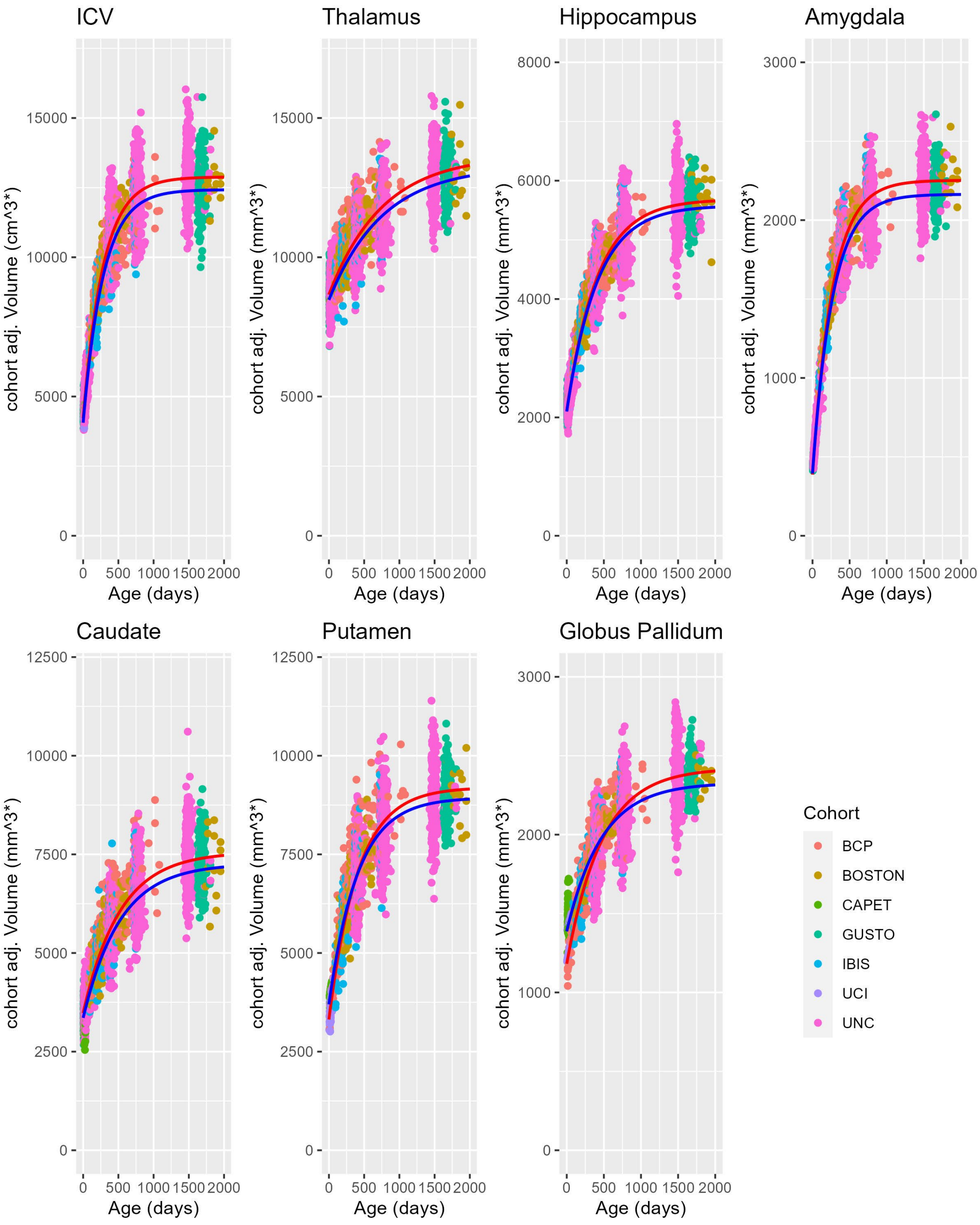

Supplementary Figure 5: Effect of low maternal education on developmental trajectories of ICV and subcortical brain structures. The trajectory of children born to mothers with lower education level (primary/secondary) is represented by blue line and that of higher education level (tertiary) by red line. Individual data points are shown in the circles and colors represent the cohorts. ICV-Intracranial Volume. BCP: Baby Connectome Project; BOSTON: Boston's Children Hospital (BCH)/Harvard Medical School; CAPET: Drakenstein Child Health Study, Cape Town; GUSTO: Growing Up in Singapore Towards healthy Outcomes, Singapore; IBIS: Infant Brain Imaging Study Network; UCI: University of California Irvine; UNC: UNC Early Brain Development Study

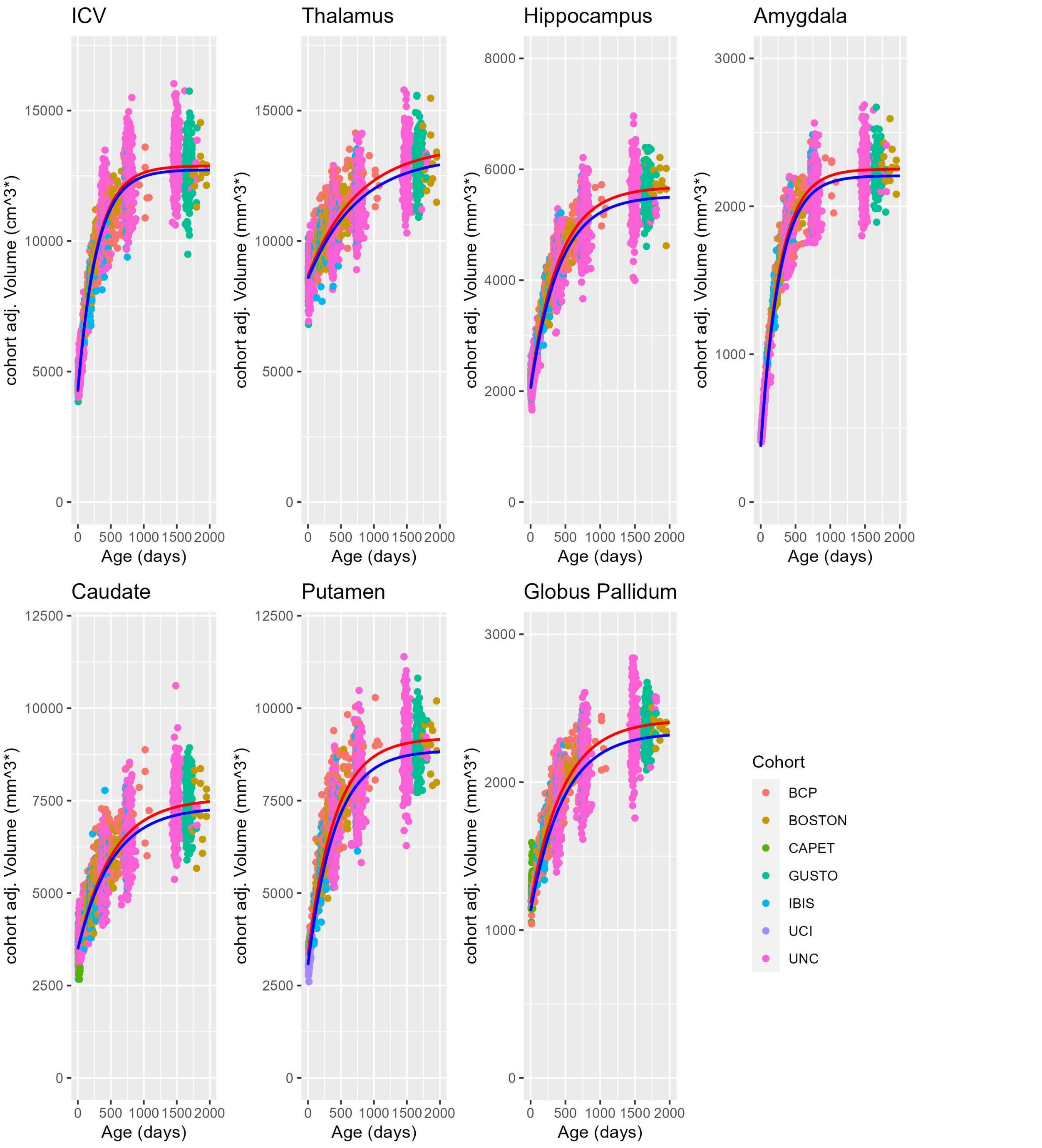

Supplementary Figure 6: Effect of low family income on developmental trajectories of ICV and subcortical brain structures. The trajectory of children coming from lower income families is represented by blue line and that of higher income families (medium/high) by red line. Individual data points are shown in the circles and colors represent the cohorts. ICV- Intracranial Volume. BCP: Baby Connectome Project; BOSTON: Boston's Children Hospital (BCH)/Harvard Medical School; CAPET: Drakenstein Child Health Study, Cape Town; GUSTO: Growing Up in Singapore Towards healthy Outcomes, Singapore; IBIS: Infant Brain Imaging Study Network; UCI: University of California Irvine; UNC: UNC Early Brain Development Study

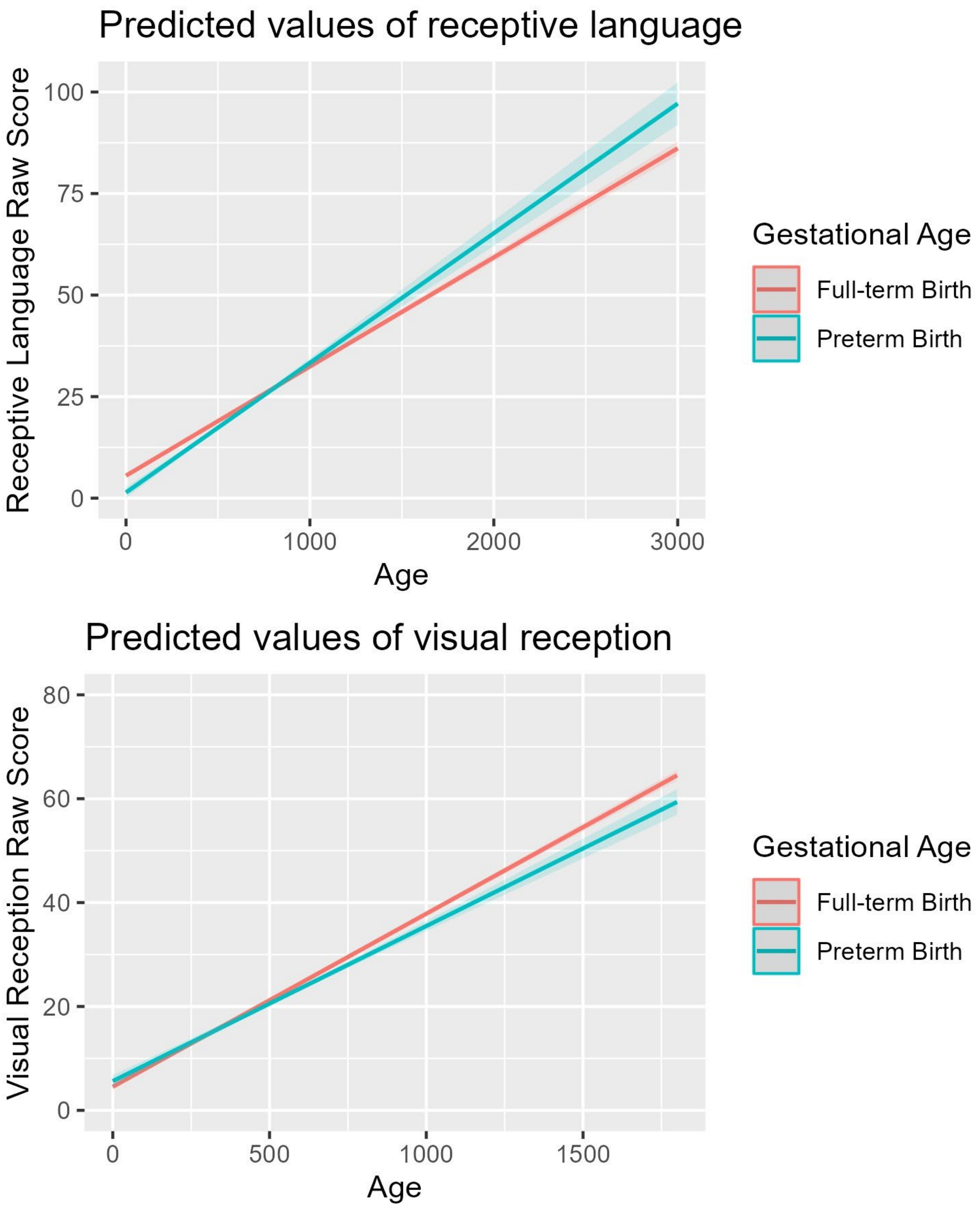

Supplementary Figure 7: Effect of gestational age on cognitive scores (A. receptive language scores B. visual reception scores). The lines represent predicted values for effect of interaction of covariates with age from the linear mixed model (Supplementary Table 9). The error bands represent 95% confidence interval for the predictions of the model. Scores for full-term born children are represented by red line and for preterm born children are represented by blue line.

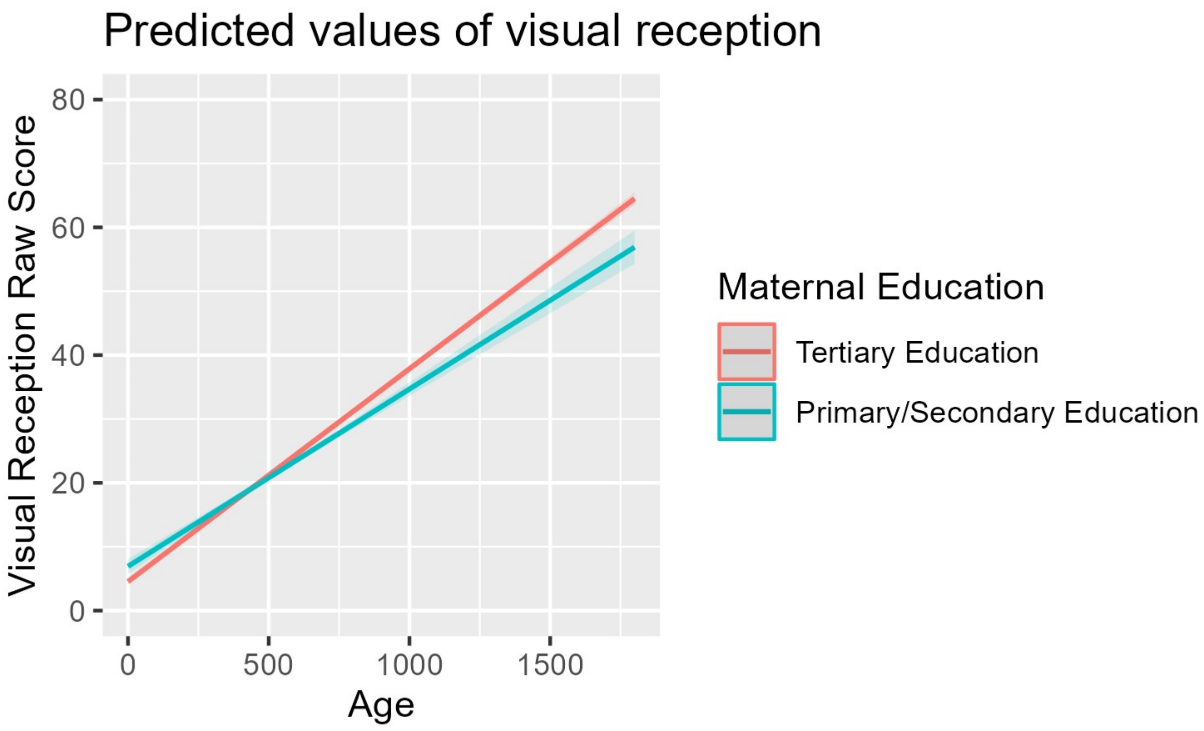

Supplementary Figure 8: Effect of maternal education on visual reception scores. The lines represent predicted values for effect of interaction of maternal education with age from the linear mixed model (Supplementary Table 9). The error bands represent 95% confidence interval for the predictions of the model. Scores for children born to mothers with tertiary education are represented by red line and children born to mothers with primary/secondary education are represented by blue line.

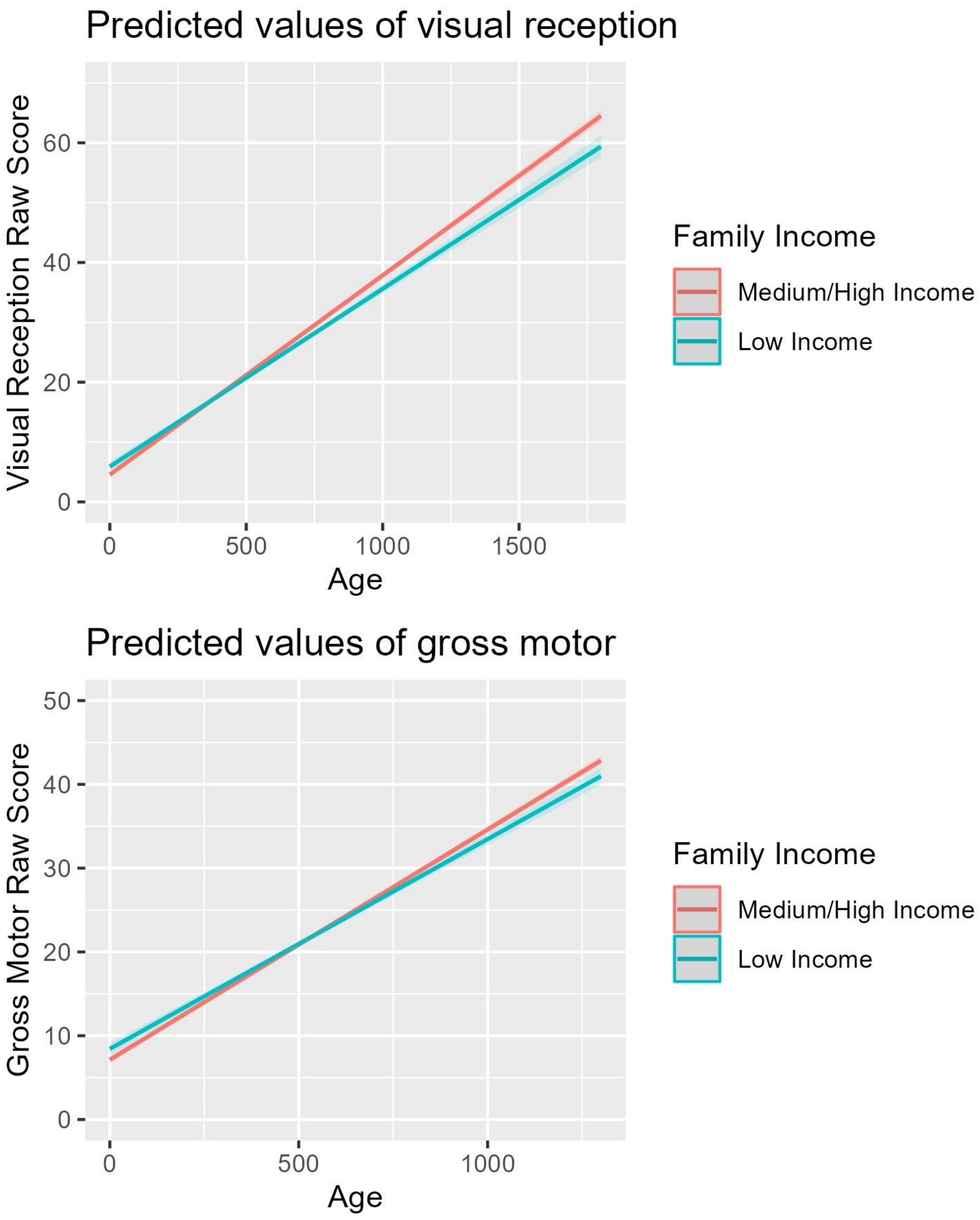

Supplementary Figure 9: Effect of family income on cognitive scores (A. visual reception scores B. gross motor scores). The lines represent predicted values for effect of interaction of covariates with age from the linear mixed model (Supplementary Table 9). The error bands represent 95% confidence interval for the predictions of the model. Scores for children from medium/high income families are represented by red line and from low-income families are represented by blue line.

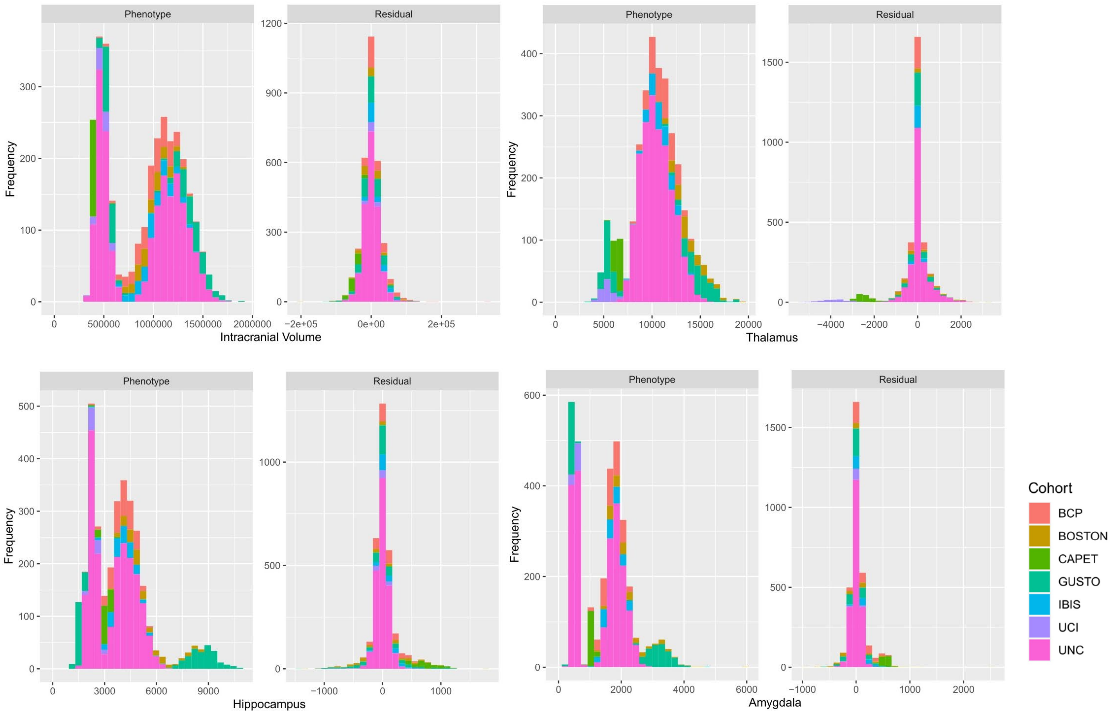

Supplementary Figure 10: Histogram of volumes (ICV, Thalamus, Hippocampus, and Amygdala) before and after modelling age, covariates, cohort, and cohort-specific error variances. BCP: Baby Connectome Project, BOSTON: Boston's Children Hospital (BCH)/Harvard Medical School; CAPET: Drakenstein Child Health Study, Cape Town; GUSTO: Growing Up in Singapore Towards healthy Outcomes, Singapore; IBIS: Infant Brain Imaging Study Network; UCI: University of California Irvine; UNC: UNC Early Brain Development Study

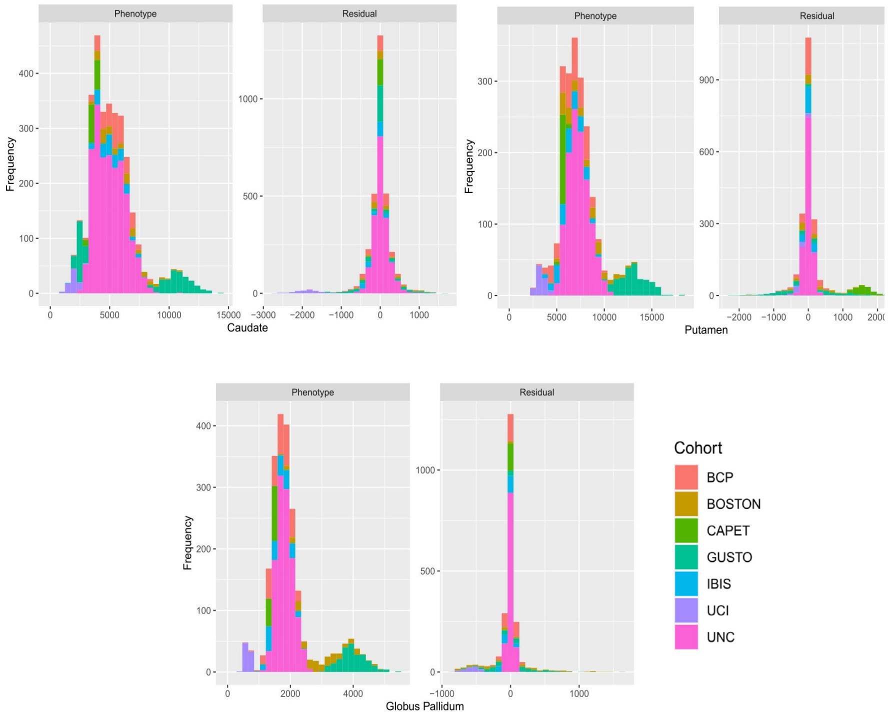

Supplementary Figure 11: Histogram of volumes (Caudate, Putamen and Pallidum) before and after modelling age, covariates, cohort, and cohort-specific error variances. BCP: Baby Connectome Project, BOSTON: Boston's Children Hospital (BCH)/Harvard Medical School; CAPET: Drakenstein Child Health Study, Cape Town; GUSTO: Growing Up in Singapore Towards healthy Outcomes, Singapore; IBIS: Infant Brain Imaging Study Network; UCI: University of California Irvine; UNC: UNC Early Brain Development Study
