# Supplementary material for: A global multicohort study to map subcortical brain development and cognition in infancy and early childhood
Source: Nat Neurosci. 2023 Nov 23;27(1):176–86. doi: 10.1038/s41593-023-01501-6 (PMC10774128; doi:10.1038/s41593-023-01501-6)
Supplement: Supplementary file 2 — Reporting Summary [file 41593_2023_1501_MOESM2_ESM.pdf]

nature portfolio

Corresponding author(s): Rebecca Knickmeyer

Last updated by author(s): Oct 13, 2023

Reporting Summary

Nature Portfolio wishes to improve the reproducibility of the work that we publish. This form provides structure for consistency and transparency in reporting. For further information on Nature Portfolio policies, see our Editorial Policies and the Editorial Policy Checklist.

Statistics

For all statistical analyses, confirm that the following items are present in the figure legend, table legend, main text, or Methods section.

n/a	Confirmed	
☐	☒	The exact sample size (*n*) for each experimental group/condition, given as a discrete number and unit of measurement
☐	☒	A statement on whether measurements were taken from distinct samples or whether the same sample was measured repeatedly
☐	☒	The statistical test(s) used AND whether they are one- or two-sided<br>*Only common tests should be described solely by name; describe more complex techniques in the Methods section.*
☐	☒	A description of all covariates tested
☐	☒	A description of any assumptions or corrections, such as tests of normality and adjustment for multiple comparisons
☐	☒	A full description of the statistical parameters including central tendency (e.g. means) or other basic estimates (e.g. regression coefficient) AND variation (e.g. standard deviation) or associated estimates of uncertainty (e.g. confidence intervals)
☐	☒	For null hypothesis testing, the test statistic (e.g. *F*, *t*, *r*) with confidence intervals, effect sizes, degrees of freedom and *P* value noted<br>*Give P values as exact values whenever suitable.*
☒	☐	For Bayesian analysis, information on the choice of priors and Markov chain Monte Carlo settings
☒	☐	For hierarchical and complex designs, identification of the appropriate level for tests and full reporting of outcomes
☐	☒	Estimates of effect sizes (e.g. Cohen's *d*, Pearson's *r*), indicating how they were calculated

Our web collection on statistics for biologists contains articles on many of the points above.

Software and code

Policy information about availability of computer code

Data collection	No software as used for data collection
Data analysis	The statistical analysis was performed in R v4.1.1. The packages used for data analysis are SPM8, nlme, lme4 (v1.1.31), cor (stats package 4.1.1), bootcorci (V0.0.0.9), and mediation packages (v.4.5.0). The code is made available in thegithub repository https://github.com/knickmeyer-lab/ORIGINs_ICV-and-Subcortical-volume-development-in-early-childhood

For manuscripts utilizing custom algorithms or software that are central to the research but not yet described in published literature, software must be made available to editors and reviewers. We strongly encourage code deposition in a community repository (e.g. GitHub). See the Nature Portfolio guidelines for submitting code & software for further information.

Data

Policy information about availability of data

All manuscripts must include a data availability statement. This statement should provide the following information, where applicable:

- Accession codes, unique identifiers, or web links for publicly available datasets
- A description of any restrictions on data availability
- For clinical datasets or third party data, please ensure that the statement adheres to our policy

The data for the study came from 8 different cohorts. The data for 4 of the cohorts is deposited in the NIMH Data Archive (NDA) and can be accessed by submitting a Data Access Request to the NDA. Imaging data for twins in the EBDS cohort is available through NDA #1974 and NDA #2384 and for singletons via NDA #4314.

April 2023

Imaging data from IBIS is available via NDA #19 and NDA #2027. Imaging data for UCI is available via NDA #1890. Imaging data for BCP is available via NDA #2848. Imaging data from the cohorts HARVARD, some of IBIS and BCP will also be made available through NDA#3905.The cognitive data from all cohorts and imaging data for the other cohorts (GUSTO, DCHS, Max Planck, Boston Children's Hospital/Harvard Medical school) can be available upon request to the parent cohort and pending IRB approval.

Research involving human participants, their data, or biological material

Policy information about studies with human participants or human data. See also policy information about sex, gender (identity/presentation), and sexual orientation and race, ethnicity and racism.

Reporting on sex and gender	Sex was included in all models for analyzing the influence on brain volume and cognitive trajectories. Of the overall sample size of 2108 individuals, 1102 were males and 1006 were females.
Reporting on race, ethnicity, or other socially relevant groupings	Maternal ethnicity distribution among the participants is provided in the supplementary table 1.
Population characteristics	Overall, the imaging cohort included 2,108 children with a total of 3,607 observations. The age-range of acquired data spans 5–2,250 postnatal days. Cognitive scores data were available within range 75 – 2,963 days. 52.3% of the participants were male and 47.7% were female.
Recruitment	Recruitment was done by the parent cohorts.
Ethics oversight	Each project was approved by their respective local review board and informed consent was obtained from parents/legal guardian and children prior to data collection. The reviewing organizations include Michigan State University, USA; Max Planck Institute for Human Cognitive and Brain Sciences, Germany; National University of Singapore, Singapore; University of Cape town, South Africa; University of North Carolina, Chapel Hill, USA; University of California, Irvine, USA; Boston's Children Hospital, USA

Note that full information on the approval of the study protocol must also be provided in the manuscript.

Field-specific reporting

Please select the one below that is the best fit for your research. If you are not sure, read the appropriate sections before making your selection.

☒ Life sciences ☐ Behavioural & social sciences ☐ Ecological, evolutionary & environmental sciences

For a reference copy of the document with all sections, see nature.com/documents/nr-reporting-summary-flat.pdf

Life sciences study design

All studies must disclose on these points even when the disclosure is negative.

Sample size	Sample size was determined based on the availability of data that had all the relevant information from each of the cohorts.
Data exclusions	The individual cohorts had data exclusion criteria which are described in the supplementary section. We analyzed all the data that was send in.
Replication	The whole sample was randomly split into two folds, and we replicated the analysis (volume trajectory, development of cognitive and motor scores, correlation analysis) 100 times. We have reported the proportion of times both the folds showed same direction of effect and proportion of times where the results from both folds were of the same sign and significant, which is a more stringent approach. The results of the replication analysis are similar to findings in the main analysis. However, some of the associations are less robust and coul be due to lower effect sizes and smaller sample sizes in the replication analysis.
Randomization	As this was an observational study, randomization does not apply.
Blinding	As this was an observational study, blinding does not apply.

Reporting for specific materials, systems and methods

We require information from authors about some types of materials, experimental systems and methods used in many studies. Here, indicate whether each material, system or method listed is relevant to your study. If you are not sure if a list item applies to your research, read the appropriate section before selecting a response.

Materials & experimental systems

n/a	Involved in the study
☒	☐ Antibodies
☒	☐ Eukaryotic cell lines
☒	☐ Palaeontology and archaeology
☒	☐ Animals and other organisms
☒	☐ Clinical data
☒	☐ Dual use research of concern
☒	☐ Plants

Methods

n/a	Involved in the study
☒	☐ ChIP-seq
☒	☐ Flow cytometry
☐	☒ MRI-based neuroimaging

Magnetic resonance imaging

Experimental design

Design type: This is an observational study using structural MRI data

Design specifications: As this was an observational study, number of blocks, trials or experimental units per session and/or subject does not apply.

Behavioral performance measures: This study used structural MRI measures. No tasks performed in scanner; we do integrate subcortical volume measures with cognitive development assessed with the Mullen Scales of Early Learning

Acquisition

Imaging type(s): Structural

Field strength: Primarily 3T, one site used 1.5T

Sequence & imaging parameters: Varies by site, full details are provided in supplementary tables 2 and 3 and 4

Area of acquisition: whole brain

Diffusion MRI: ☐ Used ☒ Not used

Preprocessing

Preprocessing software: Varies by site, full details are provided in the cohort characteristics in methods section and in Supplementary Table 4

Normalization: Varies by site, full details are provided in the cohort characteristics in methods section and in Supplementary Table 4

Normalization template: Varies by site, full details are provided in the cohort characteristics in methods section and in Supplementary Table 4

Noise and artifact removal: Varies by site, full details are provided in the cohort characteristics in methods section and in Supplementary Table 4

Volume censoring: No volume censoring

Statistical modeling & inference

Model type and settings: To map longitudinal brain development, we fitted (mixed-effects) subject-specific non-linear longitudinal growth curves to ICV and subcortical structures (thalamus, amygdala, hippocampus, caudate, putamen, and pallidum). Our growth curve models have subject-specific intercepts (i.e., volume at birth), asymptote, and growth rate parameters. In our hierarchical model, we included effects of birth outcomes and socio-demographic factors on intercepts and asymptotes, and random effects of cohort and subject. To assess brain-cognition correlations, we used Pearsons correlation between predicted brain volumes (ICV and subcortical structures), and cognitive scores at 2 years of age.

Effect(s) tested: We tested the effects of sex, birth outcomes (preterm birth and low birth weight) and socio-demographic factors (low maternal education, low maternal income) on intercepts and asymptotes of the growth curves. o assess brain-cognition correlations, we used Pearsons correlation between predicted brain volumes (ICV and subcortical structures), and predicted cognitive scores at 2 years of age. Cognitive scores included raw scores for expressive and receptive language, visual reception, and fine and gross motor function, assessed with the Mullen Scales of Early Learning

Specify type of analysis: ☐ Whole brain ☒ ROI-based ☐ Both

Anatomical location(s): Focus was on ICV and subcortical structures (thalamus, amygdala, hippocampus, caudate, putamen, and pallidum). Processing pipelines varied by site and full details are provided in the cohort characteristics in methods section and in Supplementary Table 4

Statistic type for inference

(See Eklund et al. 2016)

neither voxel or cluster-based approaches were used in the study.

Correction

Multiple comparisons correction for 35 tests (7 volumes and 5 covariates) was applied by using Bonferroni correction with original alpha level set at 0.05 resulting in a p-value significance threshold of p=0.001.

Models & analysis

n/a	Involved in the study
☒	☐ Functional and/or effective connectivity
☒	☐ Graph analysis
☒	☐ Multivariate modeling or predictive analysis
